# Effects of Selected Nigerian Medicinal Plants on the Viability, Mobility, and Multidrug-Resistant Mechanisms in Liver, Colon, and Skin Cancer Cell Lines

**DOI:** 10.3389/fphar.2020.546439

**Published:** 2020-09-15

**Authors:** Aljawharah AlQathama, Udoamaka F. Ezuruike, Andre L. D. A. Mazzari, Ahmed Yonbawi, Elisabetta Chieli, Jose M. Prieto

**Affiliations:** ^1^ School of Pharmacy, University College London, London, United Kingdom; ^2^ Department of Pharmacognosy, Faculty of Pharmacy, Umm Al-Qura University, Makkah, Saudi Arabia; ^3^ Department of Natural Products and Alternative Medicine, Faculty of Pharmacy, King Abdulaziz University, Jeddah, Saudi Arabia; ^4^ Department of Translational Research and New Technologies in Medicine and Surgery, University of Pisa, Pisa, Italy

**Keywords:** cell viability, cell migration, multi drug resistance, tyrosinase, medicinal plants

## Abstract

Medicinal plants indicated for chronic diseases usually have good safety margins as they are intended for lifelong treatments. We hypothesized that they may provide patients with baseline protection to cancers and multidrug resistance-reversing phytochemicals resulting in successful prevention and/or adjuvant treatment of chemotherapy-resistant cancers. We selected 27 popular herbal infusions widely used in Nigeria for diabetes and studied their effects on a panel of liver (HepG2), colon (Caco2), and skin (B16-F10) cancer cells. Cytotoxicity was measured using the SRB staining assay. The 2D antimigratory effect was evaluated using an Oris™ platform. The P-glycoprotein (P-gp) efflux activity was evaluated using Rh-123 as a fluorescent probe. The inhibition of tyrosinase-mediated melanogenesis was evaluated by colorimetric enzymatic assays. Our results show that melanoma cell proliferation was strongly inhibited by *Anogeissus leiocarpus* (Combretaceae)*, Bridelia ferruginea* (Phyllanthaceae), *D. ogea* (Leguminosae), and *Syzygium guineense* (Myrtaceae) extracts (G_I50 =_ 50 µg/ml). *Alstonia boonei* (Apocynaceae), *Gongronema latifolium* (Asclepiadaceae), and *Strophanthus hispidus* (Apocynaceae) were preferentially toxic against Caco2 (GI_50 =_ 50, 5 and 35 µg/ml, respectively). The most active extracts against different drug resistance mechanisms were *B. ferruginea* (inhibition of P-gp efflux, and impairing tyrosinase activity) and *X. americana* (inhibition of P-gp efflux). *A. leiocarpus, Kaya senegalensis* (Meliaceae), *S. guineense*, and *Terminalia avicennioides* (Combretaceae) significantly inhibited B16-F10 cell migration. Lupeol, ursolic acid, quercitrin, epicatechin, gallic acid, and ellagic acid were dereplicated by HPLC and HPTLC as their bioactive phytochemicals. In conclusion, the above in-vitro activities of herbal infusions regularly consumed by Nigerian diabetic patients may either act as a baseline chemoprotection or as sensitizing agents.

## Introduction

Natural products are both the base of traditional medicine and important sources of lead molecules in drug discovery (Thomford et al., 2018). One of the main global challenges to current cancer treatment is the emergence of multidrug resistance (MDR) to chemotherapeutic drugs, defined as acquired resistance to structurally and/or functionally unrelated drugs (Viale and Draetta, 2016). It is known that many herbal extracts (traditional medicines) act as chemosensitizers or resistance modulators (Tinoush et al., 2020). A key aspect of their activity is their ability to modulate multiple survival pathways with very low toxicity (Aung et al., 2017). Despite intense research, the choice of synthetic drugs against MDR is very limited (Dallavalle et al., 2020). The search for new natural product addressing this gap offers potential new avenues. Hence, our research groups is actively screening and testing for candidates from natural sources that can modulate the function, or expression, of disease-linked ABC transporters (Syed and Coumar, 2016).

The selected 27 plants in this work are widely used for diabetes and metabolic disorders in Nigeria, the most populated African country. Most of them are easily found in herbal markets all over the country for other primary indications including both common infective and non-communicable diseases Some of them have anecdotical records of use for the treatment of cancer without in-depth studies (Ezuruike and Prieto, 2014). These traditional medicines are consumed for years with apparently little toxicity and therefore may provide with a baseline defense against cancer, by killing or preventing tumor cells to acquire new hallmarks (Pan and Ho, 2008; Hodek et al., 2009). The real impact of cancer in Nigeria was largely unknown due to either a lack of statistics or under-reporting until the creation of the Nigerian National System of Cancer Registries in 2009 (The African Cancer Registry Network; Jedy-Agba et al., 2012). Emerging figures point out that more than 100,000 patients diagnosed with cancer and 70,000 deaths occur every year. These figures mean that about 1 in 10 Nigerians develop cancer before age 75 (International Agency for Research on Cancer).

In line with the topic of this special issue, we studied the cytotoxicity of the aqueous plant extracts (infusions) toward a panel of liver (HepG2), colon (Caco2) and skin (B16-F10) cancer cells as well as their effects upon selected mechanisms of drug resistance in cancer cells, namely, the efflux of xenobiotics by P-gp (Ozben, 2006), and the ability to inhibit tyrosinase-mediated melanogenesis and melanoma migration capacity (Pinon et al., 2011). Finally, we have proceeded to the identification of potential bioactive metabolites contributing to the effects of these plant extracts.

## Materials and Methods

### Plant Material

Plant materials were either bought in markets or collected from different regions in Nigeria in November 2012 by one of the authors (U.F. Ezuruike). **Table 1** summarizes their botanical details and precedence. Identification of collected plant samples collected in the north was done by Mr. Ibrahim Muazzam, an ethnobotanist and staff of the Department of Medicinal Plant Research & Traditional Medicine, National Institute for Pharmaceutical Research & Development (NIPRD). Plant samples collected in the south were verified by staff of the Forestry Research Institute of Nigeria (FRIN) in Ibadan. Vouchers were deposited in the Department of Pharmaceutical and Biological Chemistry, University College London School of Pharmacy, United Kingdom. Voucher numbers are provided in **Supplementary materials**.

**Table 1:** Summary of the botanical origin and extraction methods of the plant extracts.

Sample ID	Plant name	Family	Plant Part	Place of collection/purchase	Extraction method	Yield (%w/w)
AB	*Alstonia boonei* De Wild.	Apocynaceae	Stembark	Herbal market, Mushin	Decoction	5.03
AS	*Annona senegalensis* Pers.	Annonaceae	Stembark	Forest, Agbede	Decoction	7.10
AL	*Anogeissus leiocarpa* (DC.) Guill. & Perr.	Combretaceae	Stembark	Herbal shop, Abuja	Decoction	6.21
AD	*Anthocliesta djalonensis* A.Chev.	Gentianaceae	Stembark	Forest, Agbede	Decoction	3.05
AR	*Aristolochia repens* Mill.	Aristolochiaceae	Stem	Herbal market, Mushin	Infusion	18.11
BF	*Bridelia ferruginea* Benth.	Phyllanthaceae	Stembark	Herbal market, Ibadan	Decoction	75.86
CS	*Cassia sieberiana* DC.	Leguminosae	Root	Forest, Agbede	Decoction	10.27
CF	*Cassytha filiformis* L.	Lauraceae	Whole plant	NIPRD, Abuja	Decoction	12.66
DO	*Daniellia ogea* (Harms) Holland	Leguminosae	Stembark	Forest, Agbede	Decoction	6.05
GL	*Gongronema latifolium* Benth.	Apocynaceae	Leaves	Herbal market, Mushin	Infusion	8.37
ID	*Isoberlinia doka* Craib & Stapf	Leguminosae	Stem	Herbal market, Zaria	Decoction	4.69
KI	*Khaya ivorensis* A.Chev.	Meliaceae	Stembark	Herbal market, Mushin	Decoction	7.83
KS	*Khaya senegalensis* (Desv.) A.Juss.	Meliaceae	Stembark	Forest, Agbede	Decoction	5.02
MO	*Moringa oleifera* Lam.	Moringaceae	Leaves	Home garden, Imo	Infusion	35.54
MW	*Mondia whitei* (Hook.f.) Skeels	Apocynaceae	Stem	Herbal market, Mushin	Infusion	20.24
OG	*Ocimum gratissimum* L.	Lamiaceae	Leaves	Herbal market, Mushin	Infusion	17.04
PN	*Picralima nitida* (Stapf) T.Durand & H.Durand	Apocynaceae	Seeds	Herbal market, Ibadan	Infusion	23.71
RV	*Rauvolfia vomitoria* Afzel.	Apocynaceae	Stembark	Herbal market, Ibadan	Decoction	3.9
SD	*Scoparia dulcis* L.	Plantaginaceae	Whole plant	NIPRD, Abuja	Decoction	16.35
SL	*Securidaca longipedunculata* Fresen.	Polygalaceae	Root	Herbal market, Mushin	Decoction	7.49
SH	*Strophanthus hispidus* DC.	Apocynaceae	Stem	Herbal market, Ibadan	Decoction	12.2
SG	*Syzygium guineense* (Willd.) DC.	Myrtaceae	Stem	Herbal market, Zaria	Decoction	8.01
TA	*Terminalia avicennioides* Guill. & Perr.	Combretaceae	Stem	Herbal market, Zaria	Decoction	13.25
TB	*Tapinanthus bangwensis* (Engl. & K.Krause)	Loranthaceae	Leaves	Herbal shop, Imo	Infusion	32.2
TI	*Tamarindus indica* L.	Leguminosae	Stem	Herbal market, Zaria	Decoction	6.05
VA	*Vernonia amygdalina* Delile	Compositae	Leaves	Home garden, Lagos	Infusion	15.54
XA	*Ximenia americana* L.	Olacaceae	Stem	Herbal market, Zaria	Decoction	11.5

### Plant Sample Preparation and Extraction

All plant samples were air-dried for about two weeks (average temperature 30°C) to remove as much moisture as possible to prevent deterioration. Samples were then packed in freezer bags and shipped to the School of Pharmacy, University College London. Upon arrival, they were further dried in a Samas Vickers^®^ laboratory drying chamber electrically heated to 30°C with continuous ventilation. Samples were powdered using a laboratory scale mill (MF 10 Basic IKA WERKE blender) or an industrial size mill (Fritsch, Germany), depending on the part of the plant collected and the quantity of plant material.

The procedure for extraction of the plant samples (infusion or decoction) was based on the traditional use of the plants as described by the respondents (**Table 1**). For infusion, 300 ml of boiled distilled water was added to 10 g of plant material with continuous stirring for 15 min on a laboratory hot plate magnetic stirrer (IKA WERKE labortechnik electric stirrer). For decoction, 20 g of plant material was heated continuously under reflux in a round bottom flask containing 400 ml of distilled water for 1 h using an Electrothermal^®^ 3-in-1 laboratory heating mantle. All extracted plant samples were then allowed to stand until cold before filtering with a Buchner flask. All the filtered extracts were frozen in round bottom flasks and then freeze dried (Edwards Pirani 50 L Savant super modulo freeze drier) to obtain the dried extract. All dried plant extracts were then stored in 10 ml sample bottles at −20°C until needed.

For analyses and cell assays, plant stock concentrations (50 mg/ml) were prepared by dissolving crude extracts in bidistilled, microfiltered, aseptic water (MiliQ). The solutions were cold sterilized by microfiltration (Millipore disk filters, 0.22 microns) in a laminar flow cabinet into sterile microcentrifuge tubes. These aliquots were at −80°C until further testing.

### HPTLC Analyses

Plant extracts and phytochemical standards were diluted in methanol to a concentration of 50 and 1 mg/ml, respectively. A Linomat 5 (CAMAG, Switzerland) was used to apply 5 μl of the samples to HPTLC silica gel (Merck, Germany). The plates were developed using a CAMAG ADC2 automatic developing chamber. The method included 30-s pre-drying, 10 min humidity control using magnesium chloride to 48.3% relative humidity and 20 min saturation time, using saturation pads all done at 25.2°C. The mobile phase used was Chloroform-Methanol (8:2). During development, the solvent front was allowed to migrate 80 mm before a drying time of 5 min. For derivatization, we used Anisaldehyde/H_2_SO_4_. All visualization and analysis were done using CAMAG TLC visualizer both before and after derivatization. Phytochemical standards and solvents were from Sigma-Aldrich (UK).

### HPLC-DAD Analyses

Active plant samples were fingerprinted by HPLC-UV. Chromatograms were obtained in an Agilent 1100 Series (Gradient Quaternary Pump, Online degasser, Photodiode array detector) Software ChemStation. Elution conditions for phytomarkers were as previously described (Giner et al., 1993) on a Phenomenex^®^ C18 column (250 × 4.6 mm id, 5 μm). Solvent A (H2O + Acetic Acid 0.2%) and B (methanol + Acetic Acid 0.2% were mixed in gradient mode as follows: 0 min 90% A, 0 to 5 min 80% A, 5 to 45 min 50% A, 45 to 55 min 20% A; flow rate 0.8 ml/min. The injection volume, column temperature, and UV wavelength were set at 80 μl, 30°C and 254 nm, respectively. Phytochemical standards and solvents were from Sigma-Aldrich (UK).

### Cell Lines and Cell Culture

Caco-2 wild type cells (Sigma-Aldrich) were cultured in DMEM medium (GIBCO) supplemented with 10% FBS, penicillin (100 U/ml), streptomycin (100 μg/ml), and 1% non-essential amino acid (NEEA). The HepG2 and Caco2 cells were purchased from Sigma (UK). B16-F10 murine melanoma cell lines were from Professor Kostas Kostarelos (Centre for Drug Delivery Research, School of Pharmacy, UCL, UK). HepG2 cells (Sigma-Aldrich) were cultured in MEM medium (GIBCO) supplemented with 10% FBS, penicillin (100 U/ml), streptomycin (100 μg/ml). B16-F10 cells were cultured in DMEM medium (GIBCO) supplemented with 10% FBS, penicillin (100 U/ml), streptomycin (100 μg/ml). B16-F10 Cells were grown at 37°C and maintained in DMEM Glutamex (Dulbecco’s Modified Eagle Media) containing 4,500 mg/L D-glucose, l-glutamine (3.97 mM) and sodium pyruvate. The media was supplemented with 10% of heat-inactivated fetal bovine serum (FBS) (Gibco) and 1% penicillin-streptomycin antibiotic which consists of 10,000 units of penicillin and 10,000 µl of streptomycin per ml to inhibit bacterial growth. All cells were kept at 37°C in an incubator with a humidified atmosphere of 5% CO_2_.

### Antiproliferation Assays

The NCI protocol for SRB staining was followed as previously described (Skehan et al., 1990; National Cancer Institute, 2020). Briefly, HepG2, Caco-2, and B16-F10 cells were cultured in 96-well tissue culture plates by adding 200 μl/well of a suspension of 2 × 10^4^, 1 × 10^4^, and 2 × 10^4^ cells/well respectively. After cells reached 80% confluence the culture medium was replaced with fresh medium containing 100 μg/ml of plant extracts. After 24-h incubation with the plant extracts, media containing the samples was removed, and cells were fixed with 100 µl of cold 40% *w/v* trichloroacetic acid (TCA) solution in deionized water. The plates were incubated at 4°C for 1 h and then rinsed five times with slow running-tap water. The TCA-fixed cells were stained by adding 100 µl of SRB solution (0.4% SRB in 0.1% acetic acid) and left at room temperature for 1 h. Afterward, the plates were quickly rinsed four times with 1% acetic acid and flicked to remove unbound dye and then left to air-dry overnight. After drying completely, the protein bound SRB was solubilized by adding 100 µl of 10 mM Tris base buffer solution to each well. The plates were agitated in an orbital shaker for 30 min. The optical density was measured at 492 nm by using a microtiter plate reader (Tecan, Switzerland). Both Growth Inhibition 50% (GI_50_) and Maximum Non-Toxic Concentrations (MNTC) were calculated for each cell line.

### Rhodamine 123 Uptake Assay

Rhodamine uptake/efflux assays were conducted as previously described (Chieli et al., 2009) with minor modifications. Cells were seeded into 96-well plates for 24 h to allow for attachment. Then, the growth media was changed to serum free media. Rhodamine (5 µg/ml) was added to the wells containing 100 µg/ml of all the extracts to be tested. 20 µM verapamil was used as positive control. After 2 h incubation, cells were washed with 20 µM verapamil in PBS. Cells were lysed with 100 µl of 0.1% Triton X-100 in PBS and the plates were placed in the incubator for 15 min. 80 µl of each of the cell lysates in each well was transferred to a 96-well black plate and the fluorescence intensity of each well was measured in a Tecan^®^ plate reader (Exc-485 nm, Em-525 nm). The cellular accumulation of Rh-123 for each of the extracts was expressed as the percentage of the accumulation measured for rhodamine only, that is under control conditions.

### Tyrosinase Activity Assay

Tyrosinase inhibitory activity was assessed as previously described (Masuda et al., 2007). Briefly, L-tyrosine 2.5 mM -in PBS- and the sample- in DMSO- are mixed in a total volume of 160 µl to which 40 µl of Mushroom tyrosinase (46 units/ml) is added. After 20 min, the absorbance was measured for each well at 475 nm, then the % of Tyrosinase inhibition calculated against the control and corrected with matched blanks. All reagents were from Sigma-Aldrich (UK).

### 2D Migration Assay

The cell migration was measured using Oris^TM^ Universal Cell Migration Assembly Kit. In this assay, inserts were used to create an exclusion zone in a cell monolayer. For the migration assay, the sterile stoppers were introduced in a flat-bottom 96-well plate before starting cell seeding. B16-F10 cells were plated at a density of 7.5 × 10^4^ cells per well. After 24 h of incubation, a monolayer had been formed with an exclusion zone in the center. Afterward, the stoppers were removed manually and then the cells were incubated with the MNTC of each extract, vehicle control and hydroxyurea at (75 µM), which used as a positive control in this assay. At the end of the incubation period, the cells were fixed with 100 µl of cold 40% TCA. The plates were kept at 4°C for 1 h and rinsed with water. The cells were stained by the addition of 50 µl of trypan blue and incubated at room temperature for 1 h. Another washing step was performed, and the plate was then left to air-dry completely overnight. Measurement of zone closure was performed by digital images obtained by optical scanning (transmittance mode) by a Snapscan e50 (Agfa-Gevaert, Germany). Image J (National Health Institute, USA) was used to measure an average gap distance value and compare to the vehicle control in order to monitor the migratory capacity of the treated cells. The migration rate was calculated as follows: % Migration rate = [gap distance (day 0) − gap distance of the extract (24 h)]/[gap distance (day 0) − gap distance of vehicle control (24 h)].

### Statistics

Curve fittings, GI_50_s and IC_50_s were performed with Excel^®^ (Microsoft, Redmon) and GraphPad^®^ Instat version 3 (GraphPad Software, La Jolla, US). Significance of the results was assessed with Instat^®^ (GraphPad Software, La Jolla, US).

## Results and Discussion

### Cytotoxicity of the Extracts

Rather than calculating exact values, we classified the GI_50_ between standard doses and applied the threshold proposed by Suffness and Pezzuto (1999), which establishes that crude extracts showing an GI_50_ ≤ 100 µg/ml can be considered to be cytotoxic and selected for further studies, whereas the most promising ones are those with an GI_50_ lower than 30 µg/ml. None of the plants shown cytotoxicity toward all the cells in the panel, thus providing certain degree of specificity. The most promising cytotoxic activity was shown by *G. latifolium* against Caco2 colorectal cancer cells (GI_50_ < 10 µg/ml). Three species (*D. ogea, S. hispidus*, and *A. senegalensis*) were able to exert toxic effects on two different cell lines. Rat melanoma B16-F10 cells proliferation was strongly inhibited by *A. leiocarpus, B. ferruginea*, and *S. guineense* extracts, while *A. boonei G. latifolium, S. dulcis*, and *S. hispidus* were preferentially toxic against the human colon carcinoma Caco2 cell line. Finally, extracts from *K. ivorensis* and *S. hispidus* affected HepG2 human liver carcinoma cells only (**Table 2**).

**Table 2:** Effects of active plant extracts on cancer cell using the SRB assay.

Plant extract	Proliferation (GI_50_ µg/ml)		
	HepG2	CACO2	B16-F10
*A. boonei*	–	≈50	–
*A. leiocarpus*	–	–	≈ 50
*A. senegalensis.*	–	≈75	≈ 100
*B. ferruginea*	–	–	≈ 50
*C. filiformis*	–	–	–
*D. ogea*	≈50	–	≈ 50
*G. latifolium.*	–	≈5	–
*K. ivorensis*	≈100	–	–
*K. senegalensis*	–	–	–
*S. guineense*	–	–	≈50
*S. hispidus*	≈75	≈35	–
*T. avicennioides*	–	–	≈ 100
*T. bangwensis*	–	–	≈100
Paclitaxel	≈0.03	≈0.01	≈0.09

### Effect on Putative Drug Resistance Mechanisms

**Figure 1:**
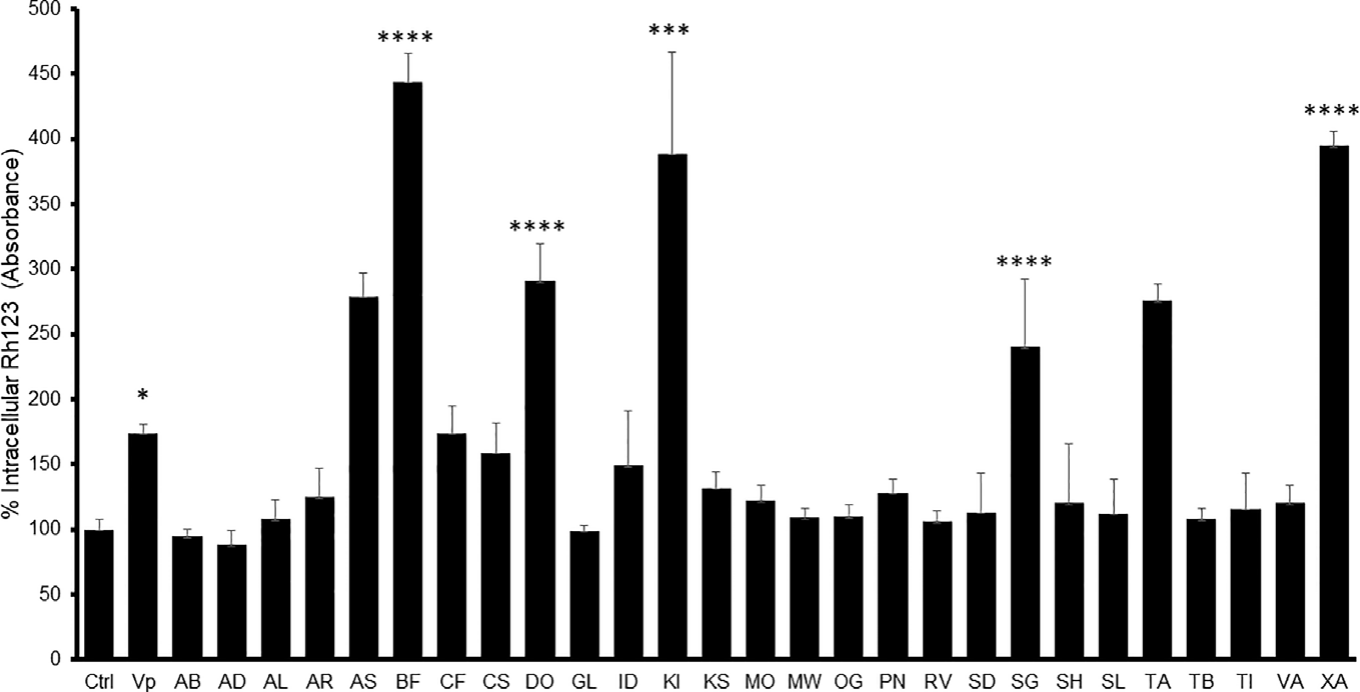
Intracellular Rh-123 concentration in Caco-2 cells after treatment with either extract (final concentration 100 µg/ml) or reference drug (Verapamil, VP). One-way ANOVA followed by Bonferroni’s post-test; Data are presented as the mean ± SD (n = 3), (****) *p <*0.0001 (***) *p* < 0.001; (*) *p* < 0.05, respectively.

The MDR-1 gene and its product, P-glycoprotein, are considered to be among the most important drug-resistance mechanisms in cancer cells (Ozben, 2006). Nine extracts obtained from *A. senegalensis*, *B. ferruginea*, *C. filiformis*, *D. ogea*, *K. ivorensis*, *S. guineense*, *T. avicennioides*, and *X. americana* produced a change in intracellular accumulation of Rh-123 which was significantly different from the control cells in Caco-2 cells (**Figure 1**) which suggests that they are acting as P-gp inhibitors.

In Caco-2 cells, the highest observed inhibitory effect was produced by *B. ferruginea* with more than 500% increase in Rh-123 accumulation compared to control cells followed by XA1 with more than 400% increase. The inhibitory effects of *B. ferruginea* and *X. americana* were thrice and twice higher than the effect of the positive control verapamil, respectively. Such high changes in intracellular Rh-123 accumulation by plant extracts are not uncommon. A similar study carried out with the stem bark extract of *Mangifera indica* produced a 1,000% increase in intracellular Rh-123 accumulation (Chieli et al., 2009).

Another emerging multidrug resistance mechanism in melanoma is melanin synthesis. Chen et al. demonstrated that melanosomes contribute to the refractory properties of melanoma cells by sequestering cytotoxic drugs and increasing melanosome-mediated drug export. Thus, preventing melanosomal sequestration of cytotoxic drugs by inhibiting the functions of melanosomes may have great potential as an approach to improving the chemosensitivity of melanoma cells (Chen et al., 2006). Furthermore, the same authors later established that the intrinsic MDR of melanoma cells is related to the ABC transporter systems -which includes P-gp- that may also play a critical role in reducing toxic melanin intermediates and metabolites (Chieli et al., 2009). The extracts that inhibited DOPA production under 100 µg/ml were *A. senegalensis* (22% ± 12), *B. ferruginea* (48% ± 10), *D. ogea* (32%± 14) and *S. guineense* (11%± 1) with Kojic acid showing an IC_50_ of 12.65 ± 3 µM in our conditions.

### Antimigratory Activity

**Figure 2:**
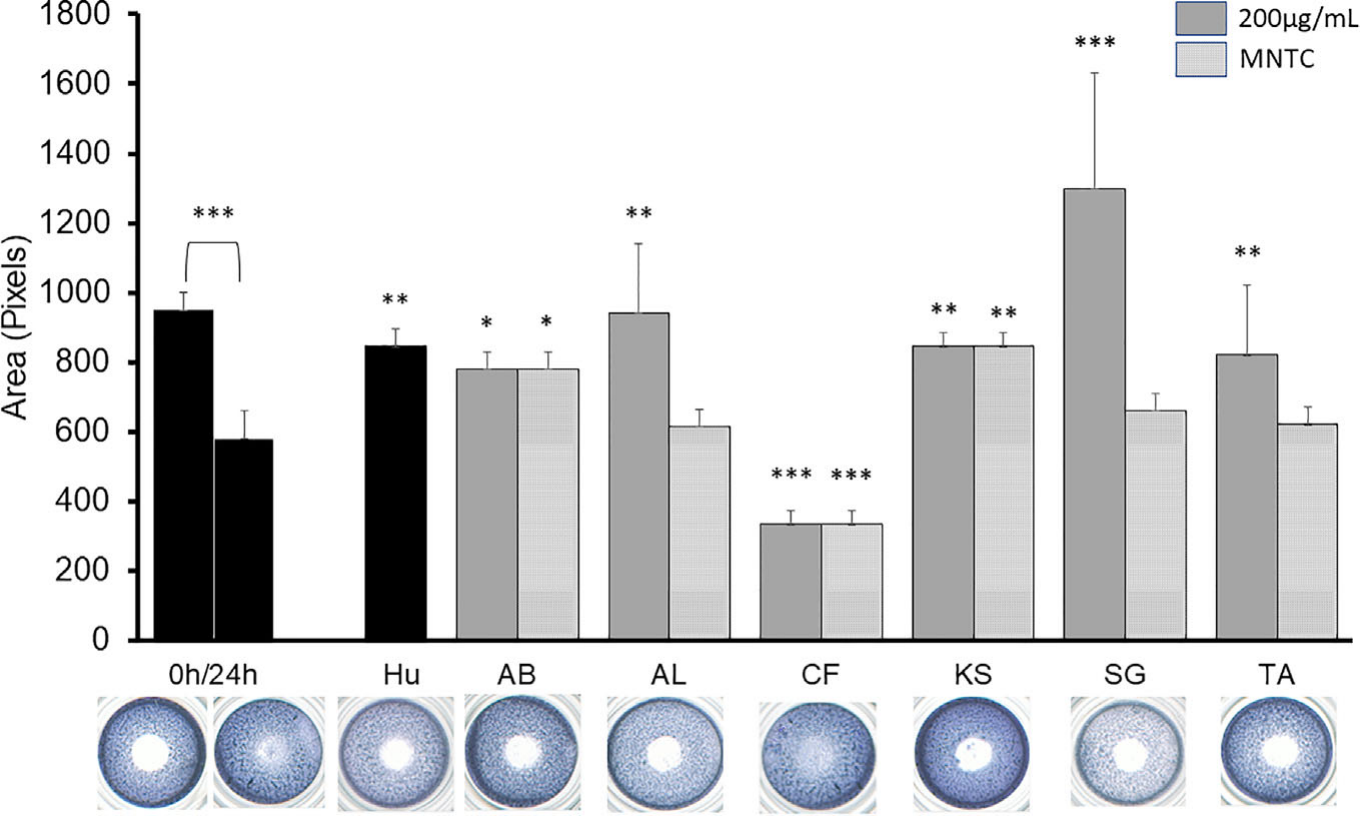
2D anti-migratory effects of the selected extracts at both GI_50_, MNTC and reference compound (HU 75 µM) on B16-F10 murine melanoma cells for 24 h. Area of the gap (measured in pixels) in a confluent cell monolayer immediately after gap generation (0 h) and at 24 h post-wounding (n = 3) with representative microscopy images for the treated gaps. The significance of inhibitory effect was determined by one-way ANOVA followed by Bonferroni’s post-test with respect to t24 control; Data are presented as the mean ± SD (n = 3), (***) *p* < 0.001; (**) *p* < 0.01; (*) *p* < 0.05, respectively.

**Figure 2** shows the effect of the active extracts on the cellular migration of the B16-F10. In vitro antimigratory activity against the highly metastatic B16-F10 cell line at 200 µg/ml was shown by *A. boonei*, *A. leiocarpus*, *K. senegalensis*, and *T. avicennioides*, while pro-migratory activity was detected for *C. filiformis.* The decoction of *S. guineense* was the most active at inhibiting migration but the cell population shown signs of toxicity. Of note, *D. ogea, A. senegalensis, B. ferruginea, M. whiteii*, and *T. bangwensis* did not exhibit a significant inhibitory effect of the 2D cell migration assay even when assayed at cytotoxic concentrations. When tested at MNTC only *A. boonei*, *K. senegalensis*, and *T. avicennioides* kept their activity.

**Figure 3:**
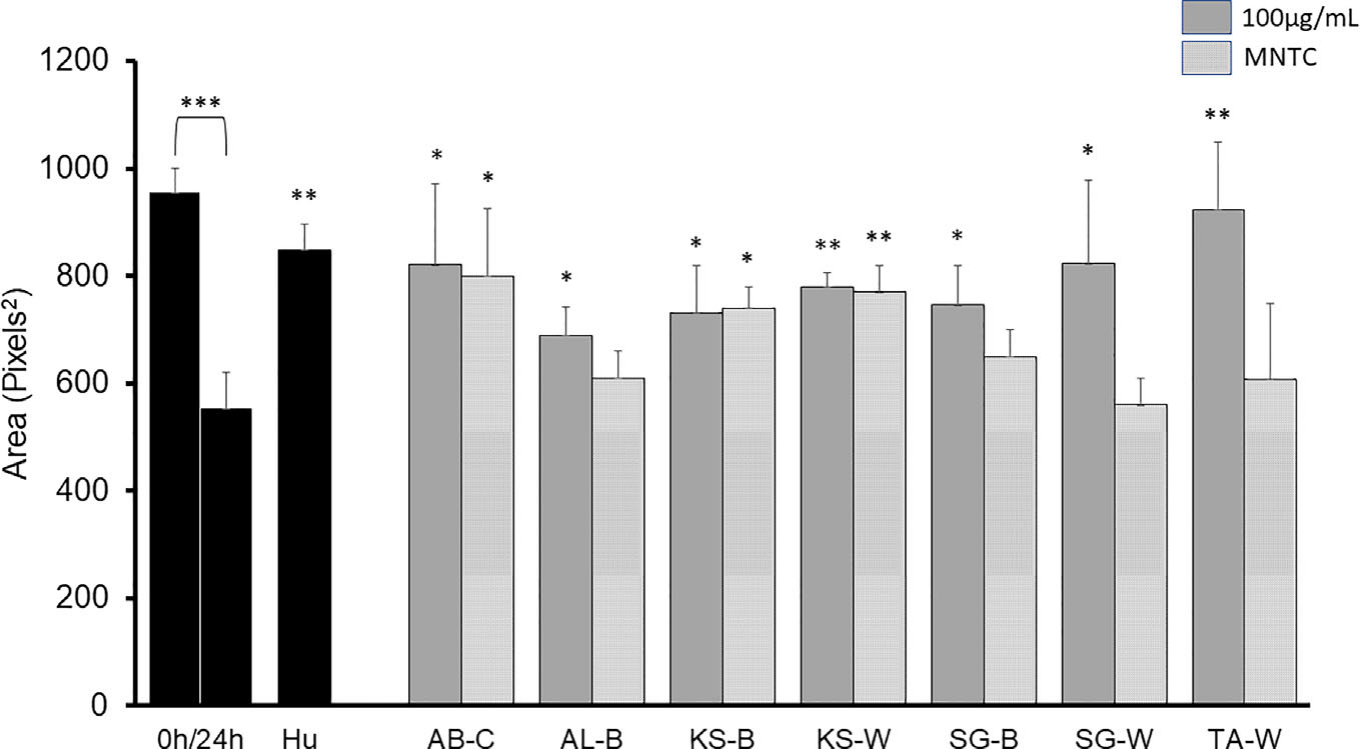
2D anti-migratory effects of fractions of active plant extracts with inhibitory effect at both GI_50_ and MNTC on B16-F10 murine melanoma cells after 24 h. Area of the gap (measured in pixels) in a confluent cell monolayer immediately after gap generation (0 h) and at 24 h post-wounding. HU: Hydroxyurea (reference compound, 75 µM); C: chloroform fraction; B: butanol fraction; W: water fraction. Data are presented as the mean ± SD (n = 3), (***) p < 0.001; (**) p < 0.01; (*) p < 0.05, respectively.

A sequential liquid/liquid extraction of the extracts was performed to obtain chloroform (C), butanol (B) and water (W) fractions with significant antimigratory effects at 100 µg/ml as shown in **Figure 3**: AB-C, AL-B, KS-B, KS-W, SG-B, SG-W, and TA-W. When tested at MNTC only AB-C, KS-B, and KS-W kept their activity intact.

### Dereplication of Known Bioactive Compounds

Screening of natural products for potential anticancer activities must always be accompanied by dereplication strategies so to avoid needless isolation of known bioactive compounds (Corley and Durley, 1994).

All extracts were chromatographed and dereplicated for Gallic acid, caffeic acid, epicatechin, epigallocatechin, catechin, vitexin rhamnoside, vitexin, rutin, ellagic acid, quercitrin, hesperidin, quercetin, luteolin, kaempferol, ursolic acid, betulinic acid, and lupeol. The resulting chromatograms are provided as supplementary materials. **Supplementary Table 1** summarizes the phenolic compounds detected in our plant samples *versus* those already reported in literature for the plant species.

**Figure 4:**
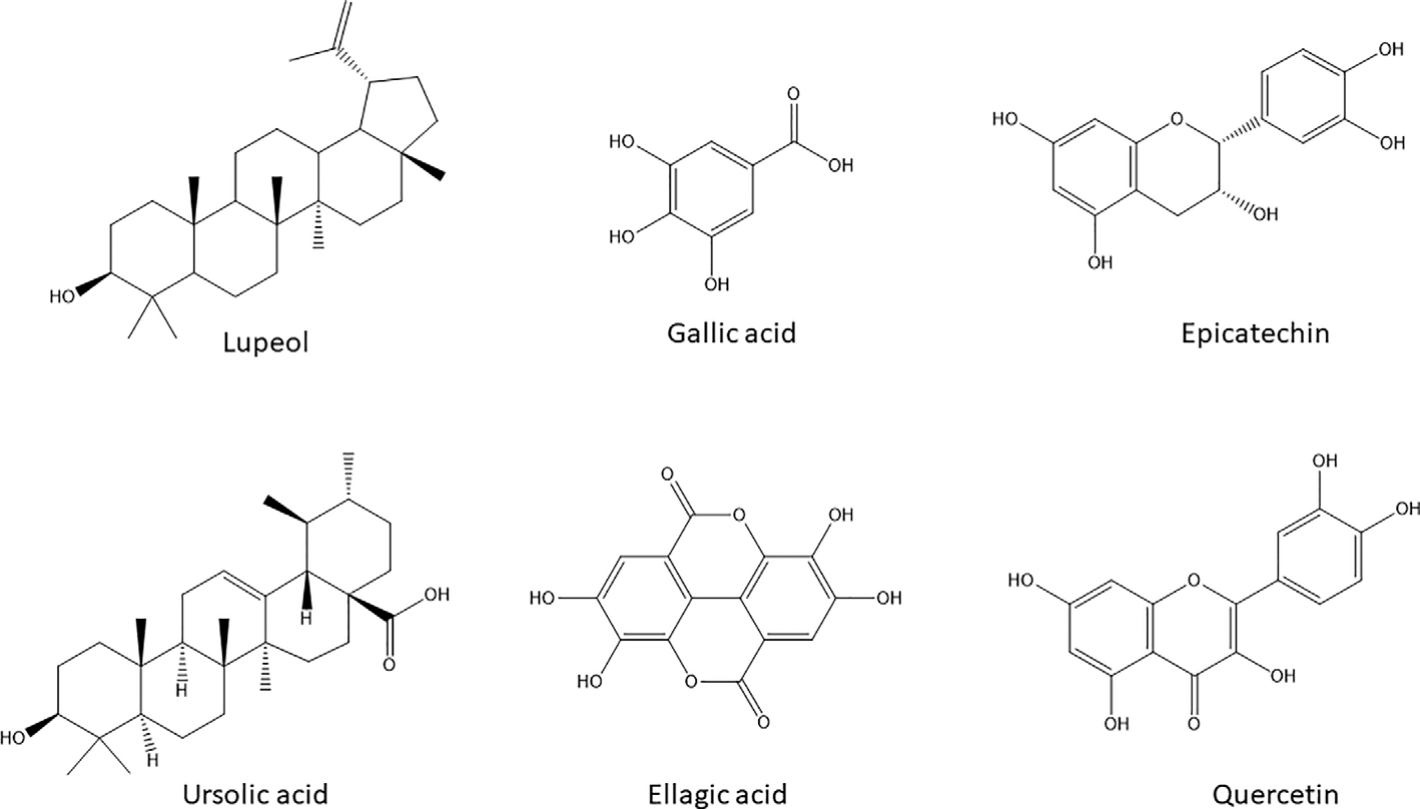
Chemical structures of the natural products with potential anticancer and antimigratory activities dereplicated by HPLC-DAD and HPTLC in the selected active extracts.

The activities of *A. boonei* may be explained by the presence of lupeol [**1**], ursolic acid [**2**] (TLC) and quercitrin [**6**] (HPLC); *A. leiocarpus* contains Epicatechin [**5**], Gallic acid [**3**], and ellagic acid [**4**] (HPLC) and *S. guineense* Gallic acid [**3**] (HPLC). Their chemical structures are presented in **Figure 4**.

Lupeol [**1**] and ursolic acid [**2**] are regarded as powerful inducers of apoptosis in cancer cells with added modulatory effects upon drug resistance mechanisms in cancer cells including P-gp activity (Yan et al., 2014). Gallic acid [**3**] besides its well- known antioxidant activity can decrease P-gp expression in HK-2 cells (Chieli et al., 2010) and directly modify its function on KB-C2 cells (Kitagawa et al., 2004). Ellagic acid [**4**], Epicatechin [**5**], and quercitrin [**6**] are also endowed with anticarcinogenic effects by inhibiting tumor cell proliferation, inducing apoptosis and inhibiting drug-resistance processes—specially efflux pumps and transporters—required for tumor growth (Tan et al., 2013; Zhang et al., 2014). Moreover, all these chemicals are inhibitors of migration of melanoma cell lines (Alqathama and Prieto, 2015).

## Conclusions

This study results demonstrated that some of these herbal medicines are endowed with significant *in vitro* cytotoxicity and inhibit MDR mechanisms. More refined studies would be necessary to ascertain the *in vivo* and/or clinical significance of such properties. In terms of direct cytotoxic effects, the aqueous extracts of G. *latifolium* and *S. hispidus* emerge as promising leads according the strictest standards (GI_50_ < 30 µg/ml, Caco2 cells). It is conceivable that traditional infusions can reach pharmacologically active levels in the gastrointestinal system. Therefore, their chronic intake by diabetic patients may provide a baseline protection against colon cancer. This warrants further *in vivo* studies to confirm if their active principles are not degraded in the stomach and can reach the colon.

On the other hand, *B. ferruginea* and *X. americana* shown the largest effects on P-gp efflux, a clinically relevant target. These herbs have additional inhibitory effects on other putative MDR mechanisms namely depletion of glutathione as per our previous work (Ezuruike et al., 2019) and impairing tyrosinase activity (as per this work), respectively. Thus, they are clear candidates for adjuvant or combination cancer therapy.

Our study also shows for first time that the following herbal extracts exert *in vitro* antimigratory activity against the highly metastatic B16-F10 cell line: *A. leiocarpus, K. senegalensis*, *S. guineense*, and *T. avicennioides*. Some of their bioactive phytochemicals (lupeol, ursolic acid gallic acid, ellagic acid, epicatechin, and quercitrin) were dereplicated by HPLC and HPTLC.

Although further studies are needed to attest the clinical relevance of our findings, we hope that this work will stimulate similar research toward establishing the clinical relevance of these herbal medicines in Nigeria.

## Data Availability Statement

The raw data supporting the conclusions of this article will be made available by the authors, without undue reservation, to any qualified researcher.

## Author Contributions

AA performed cytotoxicity assays and 2D migratory assays (B16-F10 cells). UE collected plant materials in the field, performed cytotoxicity assays (Caco2, HepG2) and P-gp assay. AY performed tyrosinase activity assays. AM and EC designed, supervised, and advised on P-gp assay. JP designed the overall work, supervised all assays, performed HPLC analyses, and wrote the manuscript.

## Funding

This research project was conducted with support from the Saudi Arabian Ministry of Education, Umm Al-Qura University (SACB Ref. UMU397) and The Commonwealth (Scholarship NGCS-2010-306).

## Conflict of Interest

The authors declare that the research was conducted in the absence of any commercial or financial relationships that could be construed as a potential conflict of interest.
