# Supplementary material for: Effects of Selected Nigerian Medicinal Plants on the Viability, Mobility, and Multidrug-Resistant Mechanisms in Liver, Colon, and Skin Cancer Cell Lines
Source: Front Pharmacol. 2020 Sep 15;11:546439. doi: 10.3389/fphar.2020.546439 (PMC7533547; doi:10.3389/fphar.2020.546439)
Supplement: Supplementary file 1 [file DataSheet_1.pdf]

Supplementary Materials

Effects of selected Nigerian medicinal plants on the viability, mobility and multidrug-resistant mechanisms in liver, colon and skin cancer cell lines.

Aljawharah AlQathama[1,2 *], Udoamaka F. Ezuruike[1], Andre L. D. A. Mazzari[1], Ahmed Yombawi[1,3], Elisabetta Chieli[4], Jose M. Prieto[1]

1. School of Pharmacy, University College London, 29-39 Brunswick Square, WC1N 1AX London, United Kingdom.
2. Department of Pharmacognosy, Faculty of Pharmacy, Umm Al-Qura University, Makkah, 21955, Saudi Arabia.
3. Department of Natural Products and Alternative Medicine, Faculty of Pharmacy, King Abdulaziz University, Jeddah, Saudi Arabia.
4. Department of Translational Research and New Technologies in Medicine and Surgery, University of Pisa, Italy.

(*) Correspondence author: aaqathama@uqu.edu.sa

Keywords: Cell viability1; Cell migration2; Multi Drug Resistance3; Tyrosinase4; Medicinal Plants5

Supplementary Materials

1 HPLC FINGERPRINTS - ANALYSIS

The elution conditions previously described in material and methods allowed for a good separation of all the fifteen phytochemical standards. The chromatogram obtained is shown below (Supplementary Figure 1S). A HPLC-UV fingerprint for each of the plant extracts (50 micrograms/mL) was also obtained under the same chromatographic conditions (Supplementary Figures 2S-27S).

Supplementary Figure 1S. HPLC-UV chromatogram of phytochemical standards (254nm)

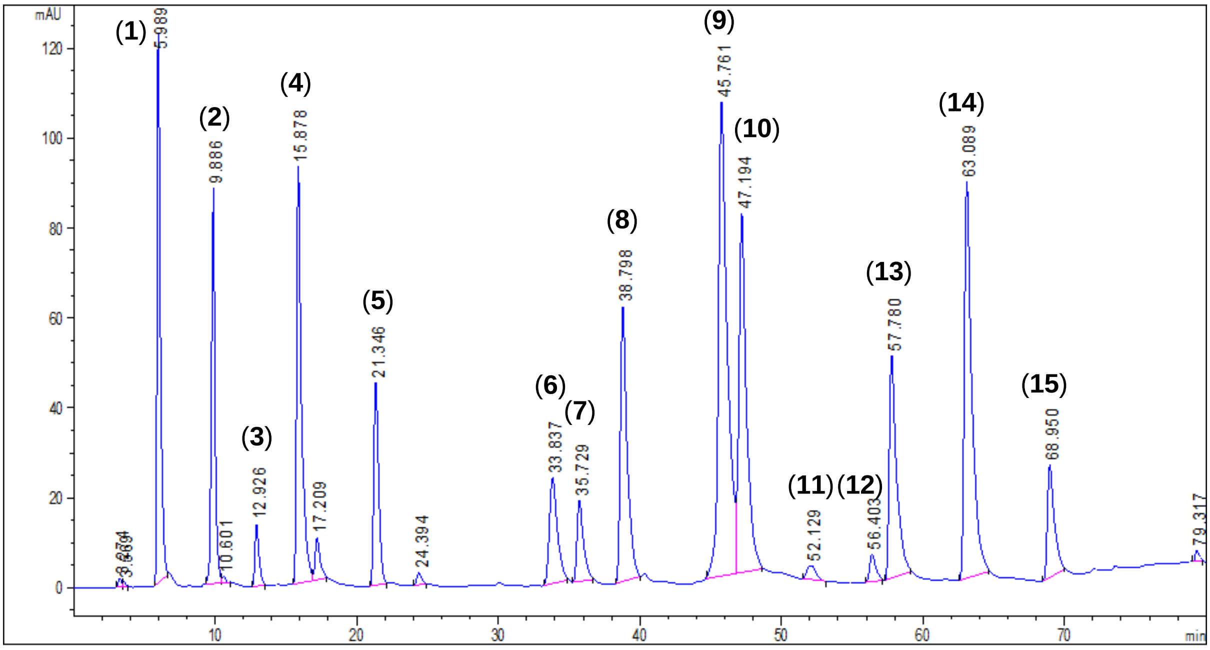

(1) Gallic acid, (2) 2,4-dihydroxybenzoic acid, (3) Caffeic acid, (4) Epicatechin, (5) Epigallocatechin, (6) Catechin, (7) Vitexin rhamnoside, (8) Vitexin, (9) Rutin, (10) Ellagic acid, (11) Quercitrin, (12) Hesperidin, (13) Quercetin, (14) Luteolin and (15) Kaempferol. Each standard was present in the cocktail at a final concentration of 1mg/ml.

Supplementary Materials

Supplementary Figure 2S. HPLC chromatogram (254 nm) of *A. boonei* (voucher no. SOP052011AB01)

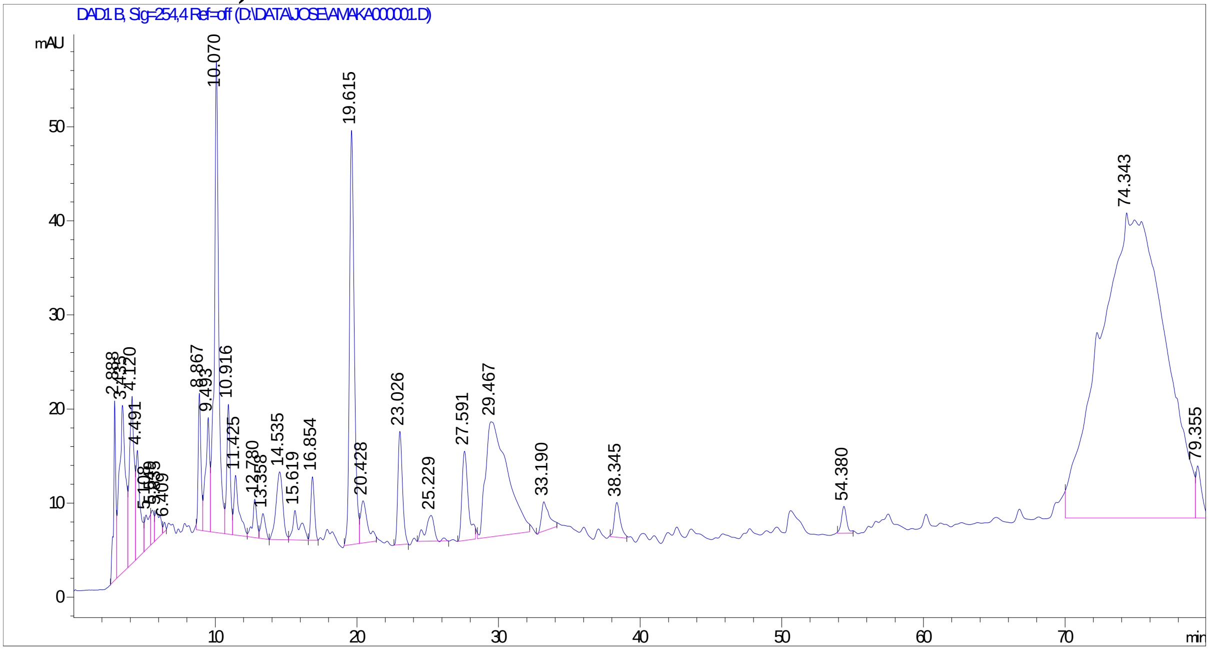

Supplementary Figure 3S. HPLC chromatogram (254 nm) of *A. leicocarpus* (voucher no. SOP052011AL01)

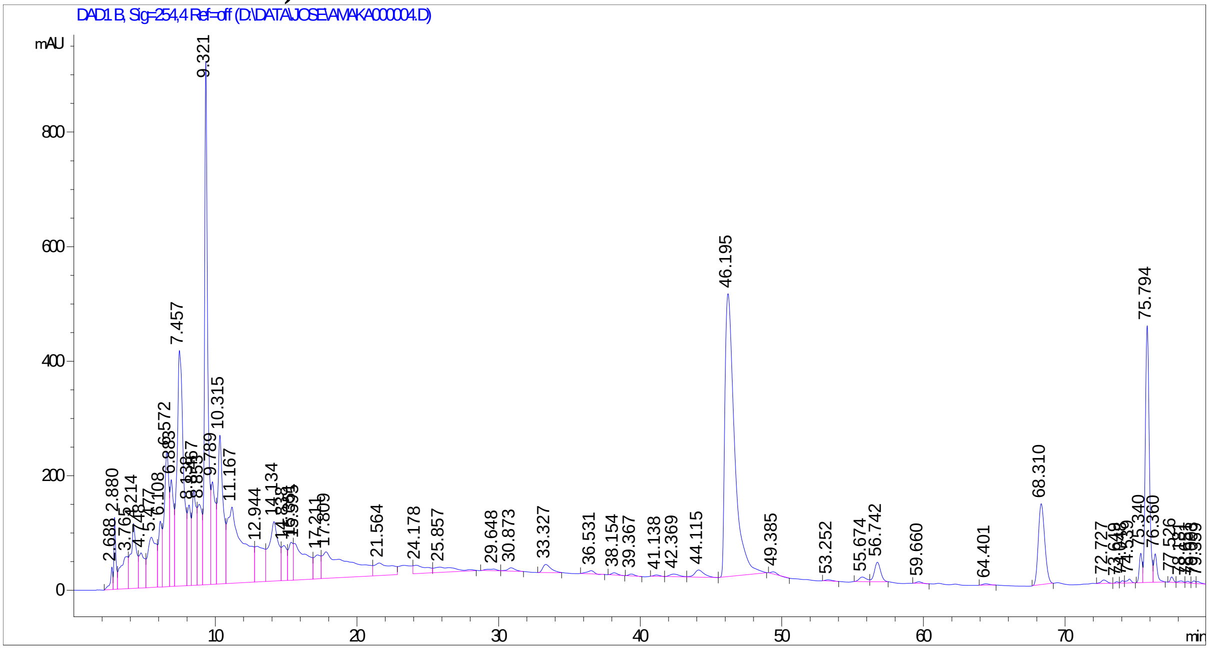

Supplementary Figure 4S. HPLC chromatogram (254 nm) of *A. senegalensis* (voucher no. SOP052011AS01)

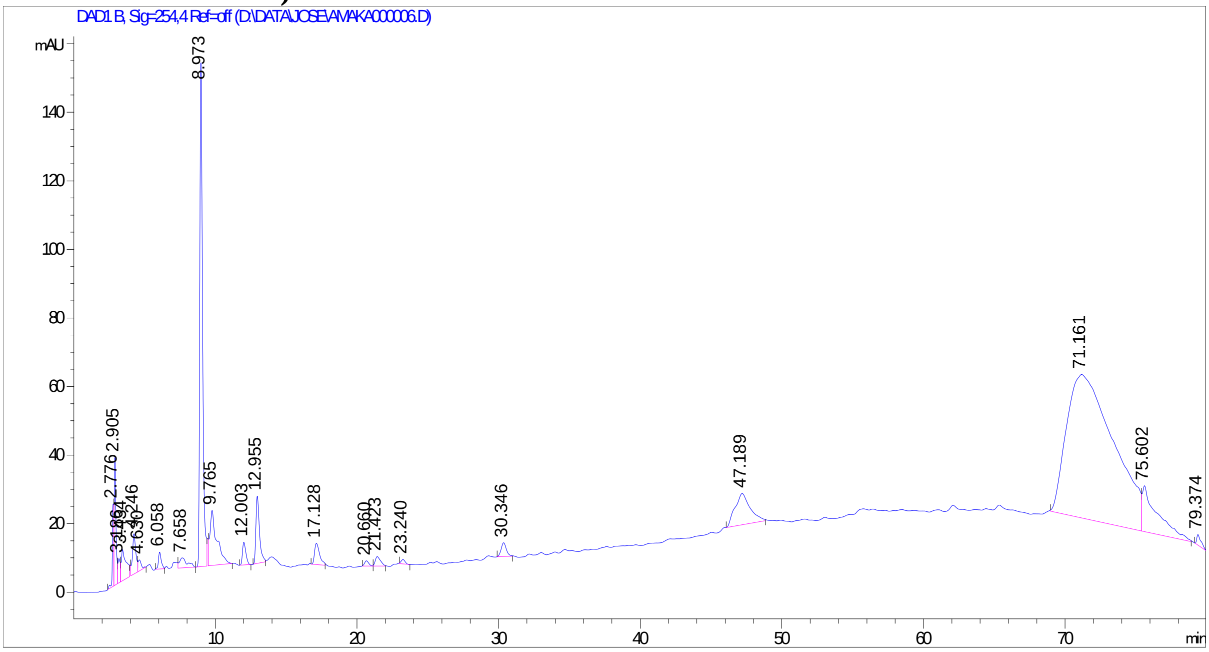

Supplementary Figure 5S. HPLC chromatogram (254 nm) of *A. djalonensis* (voucher no. SOP052011AD01)

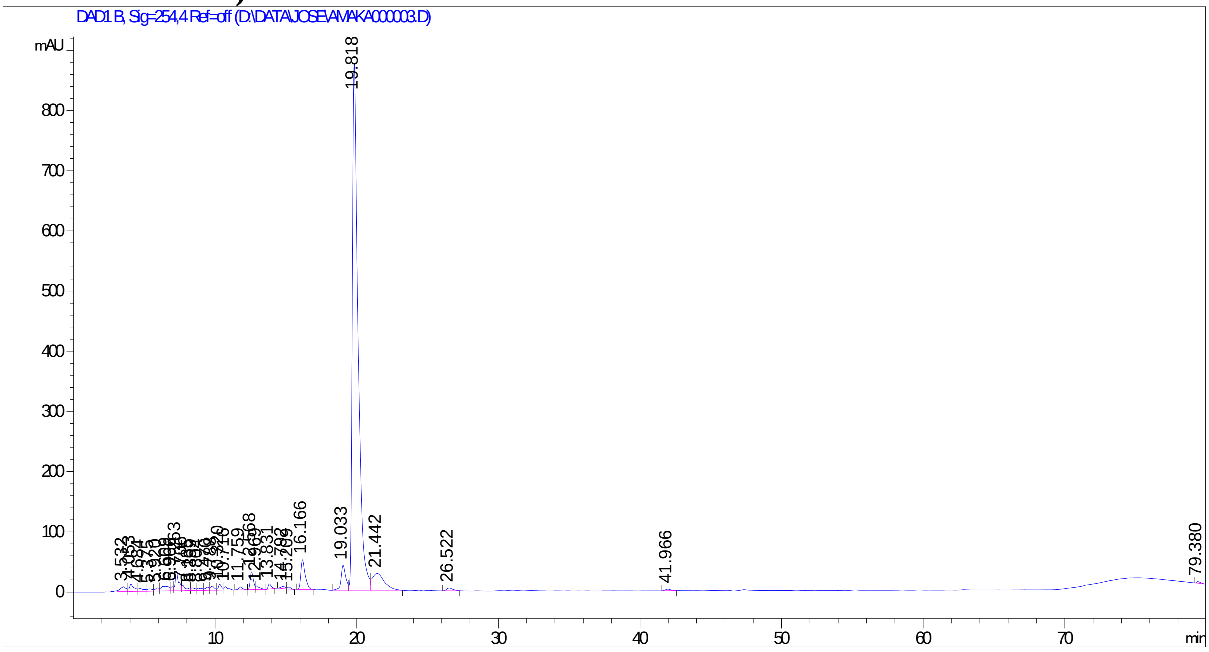

Supplementary Figure 6S. HPLC chromatogram (254 nm) of *A. repens* (voucher no. SOP052011AR01)

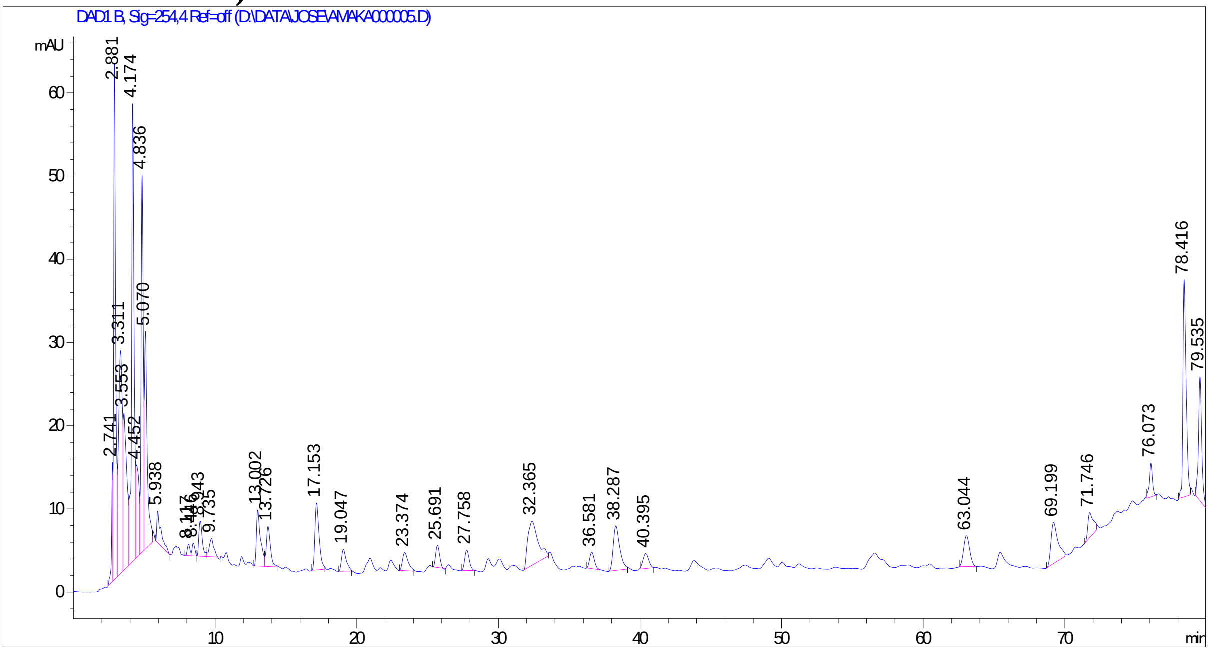

Supplementary Figure 7 S. HPLC chromatogram (254 nm) of *B. ferruginea* (voucher no. SOP052011BF01)

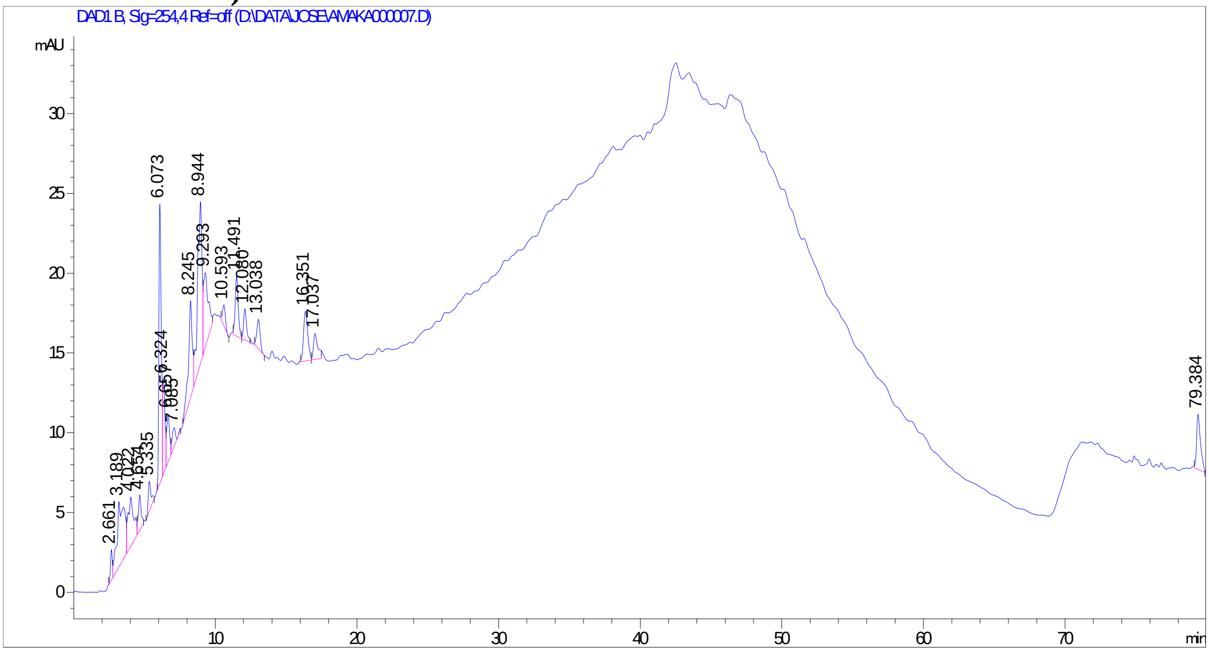

Supplementary Figure 8 S. HPLC chromatogram (254 nm) of *C. sieberiana* (voucher no. SOP052011CS01)

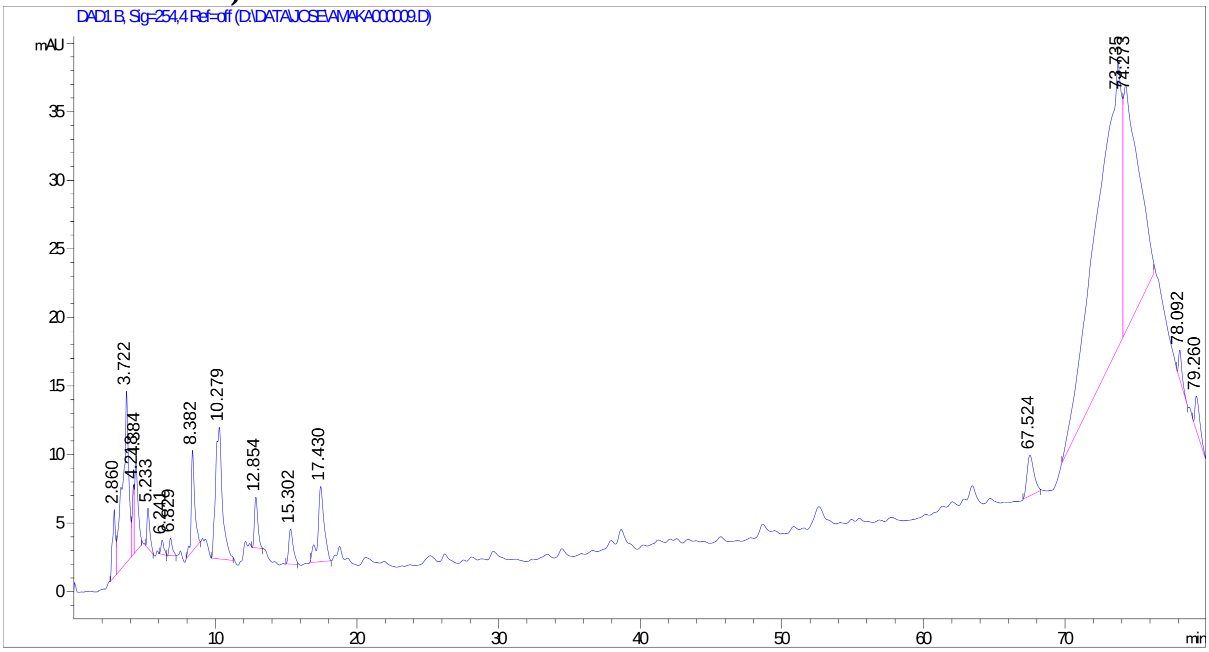

Supplementary Figure 9 S. HPLC chromatogram (254 nm) of *C. filiformis* (voucher no. SOP052011CF01)

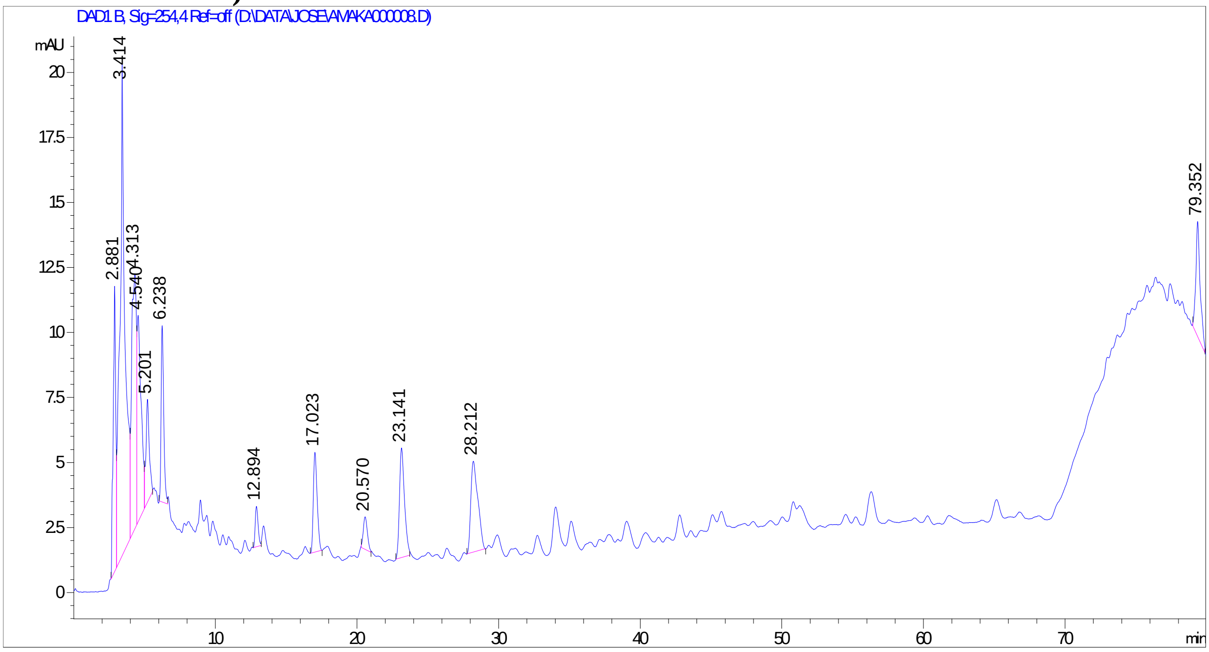

Supplementary Materials

Supplementary Figure 10S. HPLC chromatogram (254 nm) of *D. ogea* (voucher no. SOP052011DO01)

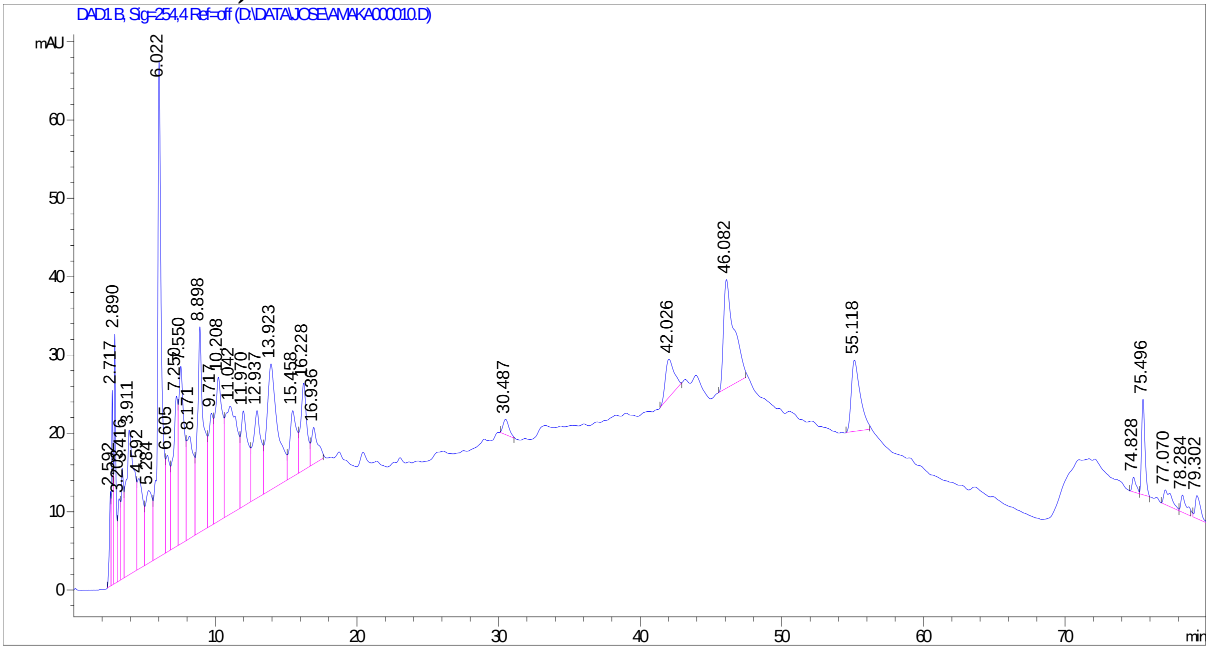

Supplementary Figure 11S. HPLC chromatogram (254 nm) of *G. latifolium* (voucher no. SOP052011GL01).

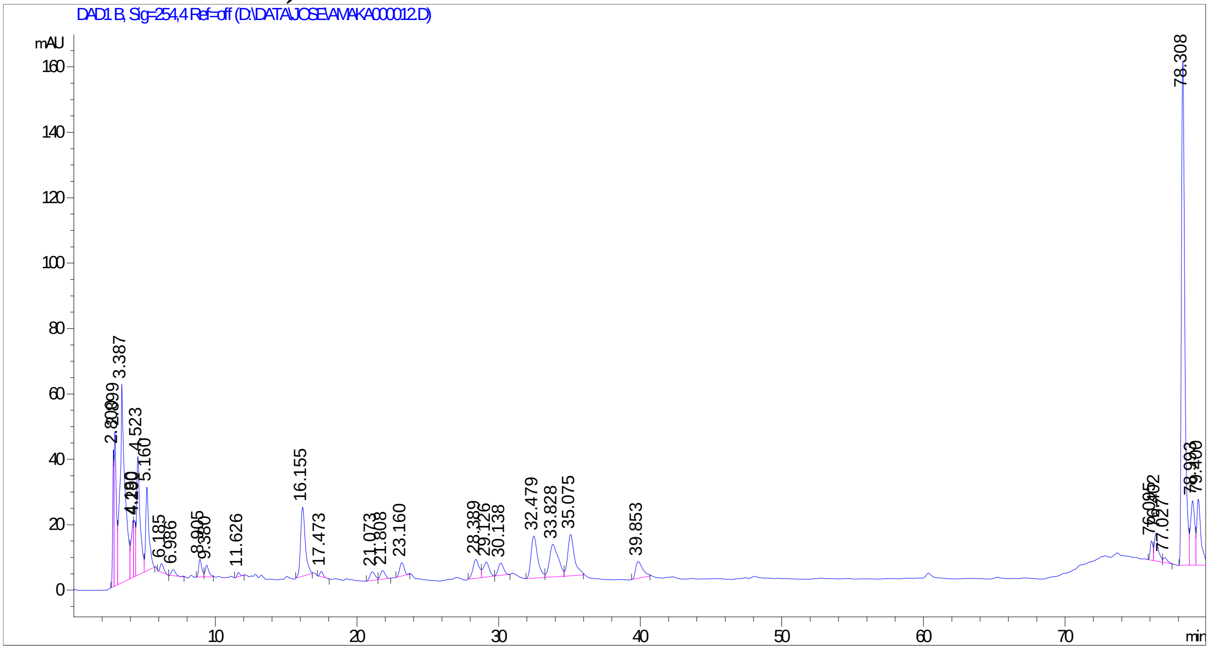

Supplementary Figure 12S. HPLC chromatogram (254 nm) of *I. doka* (voucher no. SOP052011ID01)

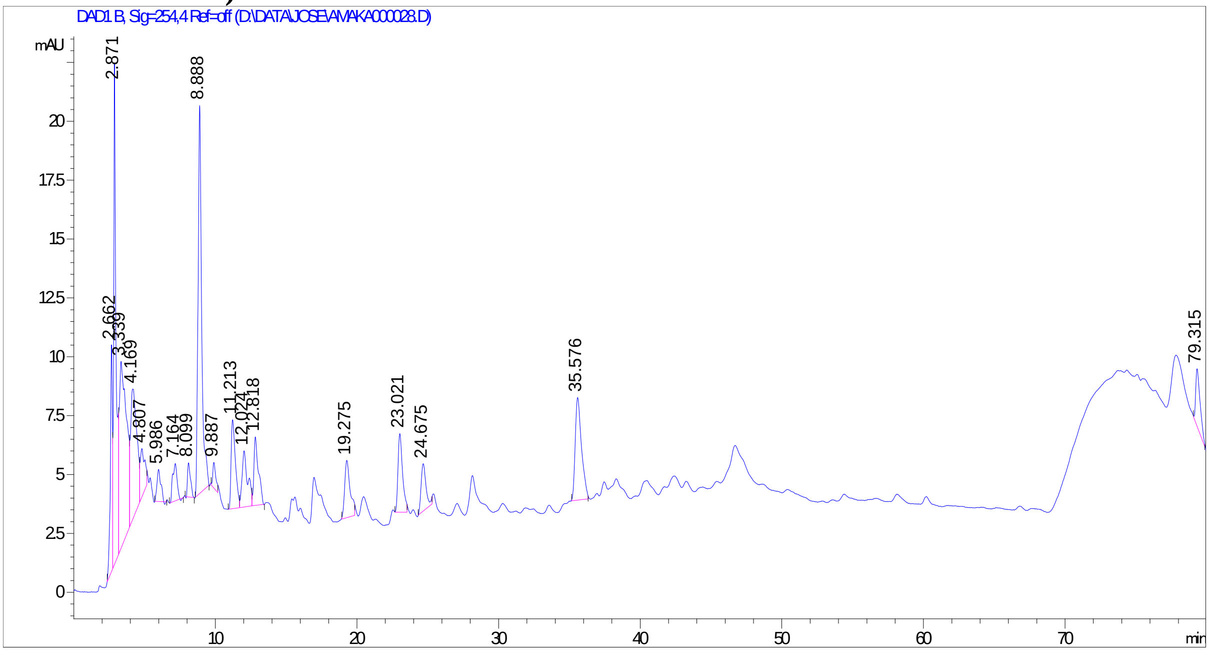

Supplementary Figure 13S. HPLC chromatogram (254 nm) of *K. ivorensis* (voucher no. SOP052011KI02)

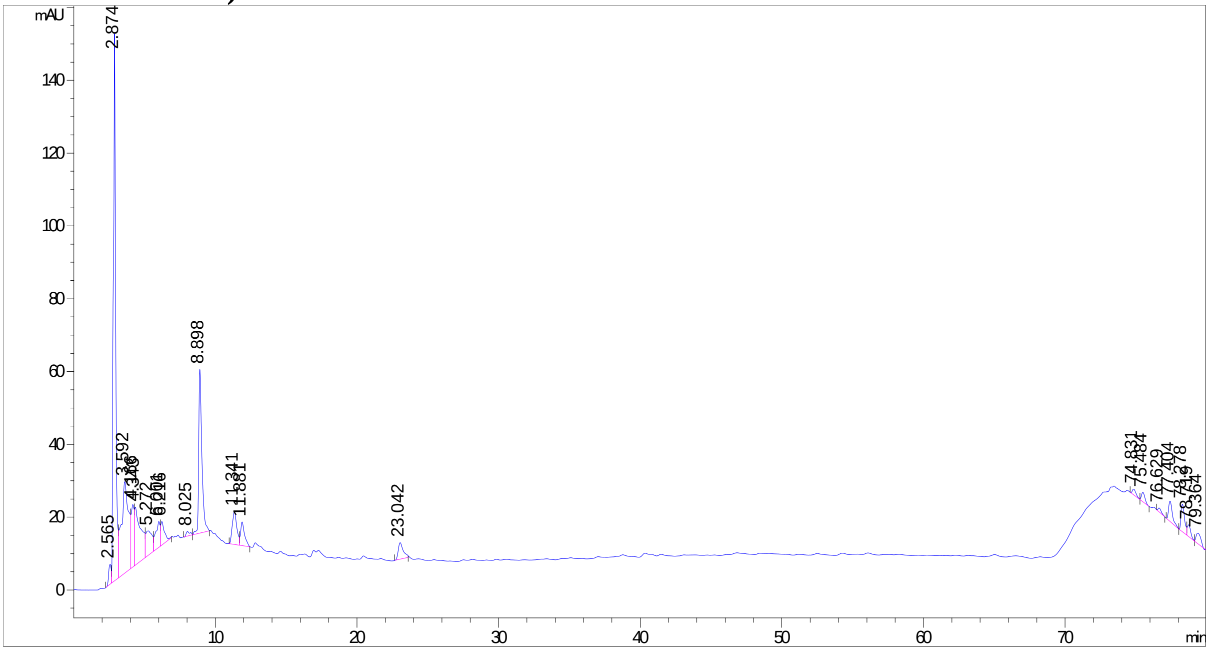

Supplementary Figure 14S. HPLC chromatogram (254 nm) of *K. senegalensis* (voucher no. SOP052011KS01)

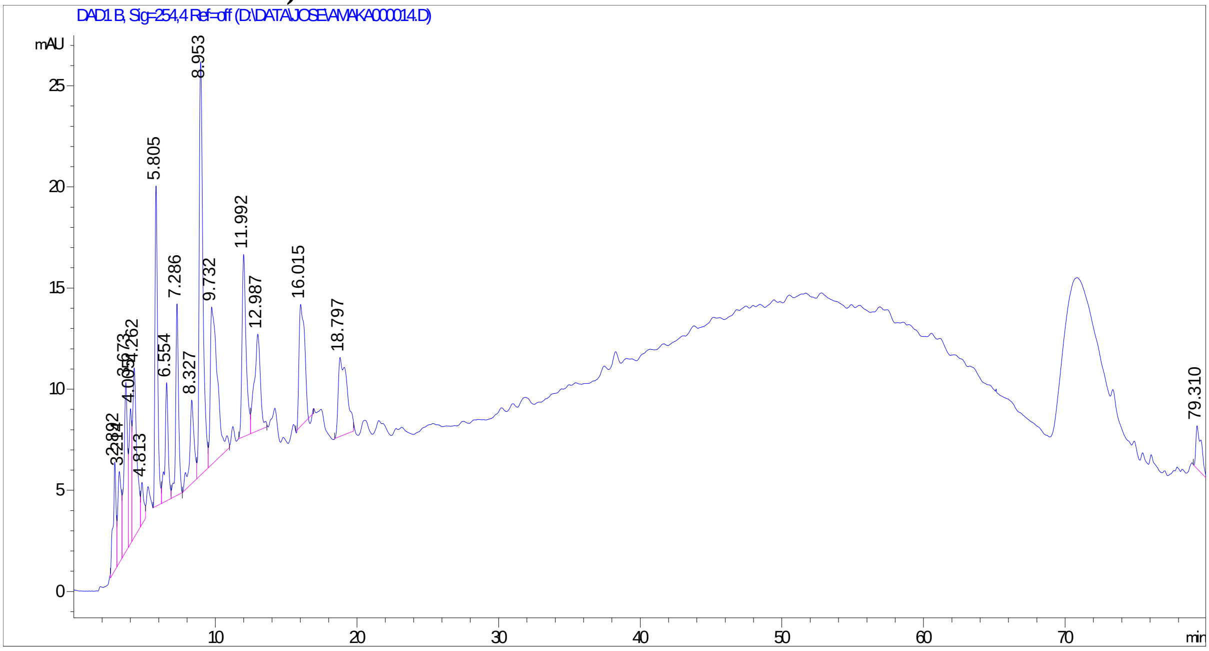

Supplementary Figure 15S. HPLC chromatogram (254 nm) of *M. whitei* (voucher no. SOP052011MW01)

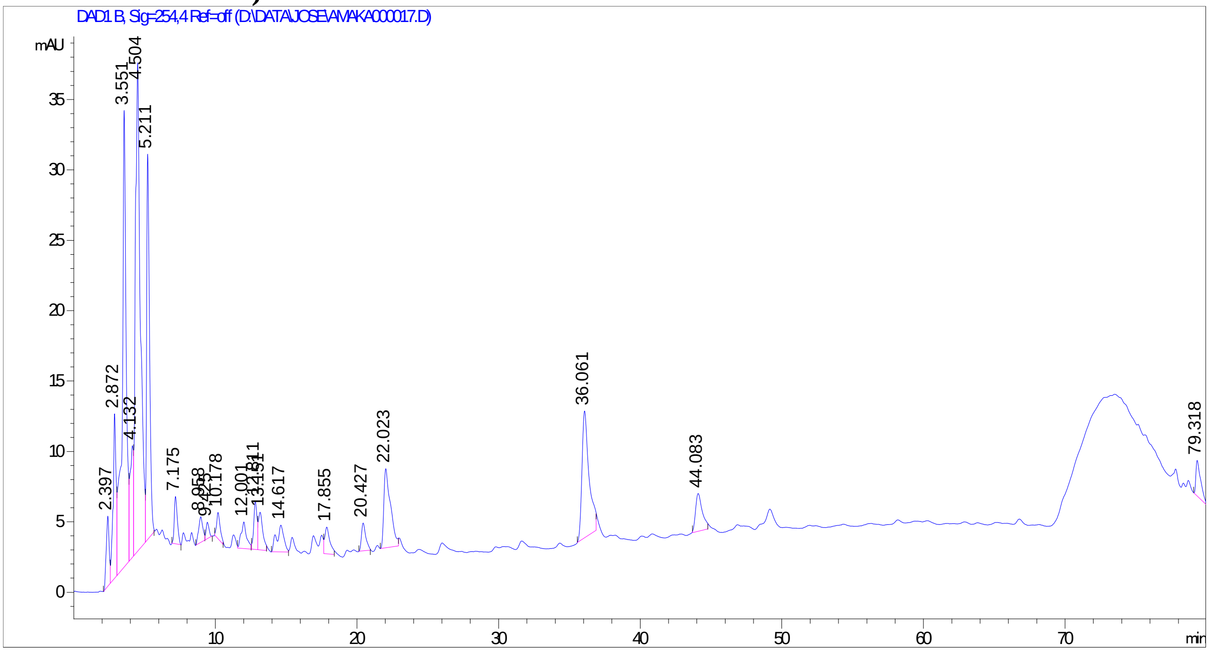

Supplementary Materials

Supplementary Figure 16S. HPLC chromatogram (254 nm) of *M. oleifera* (voucher no. SOP052011MO01)

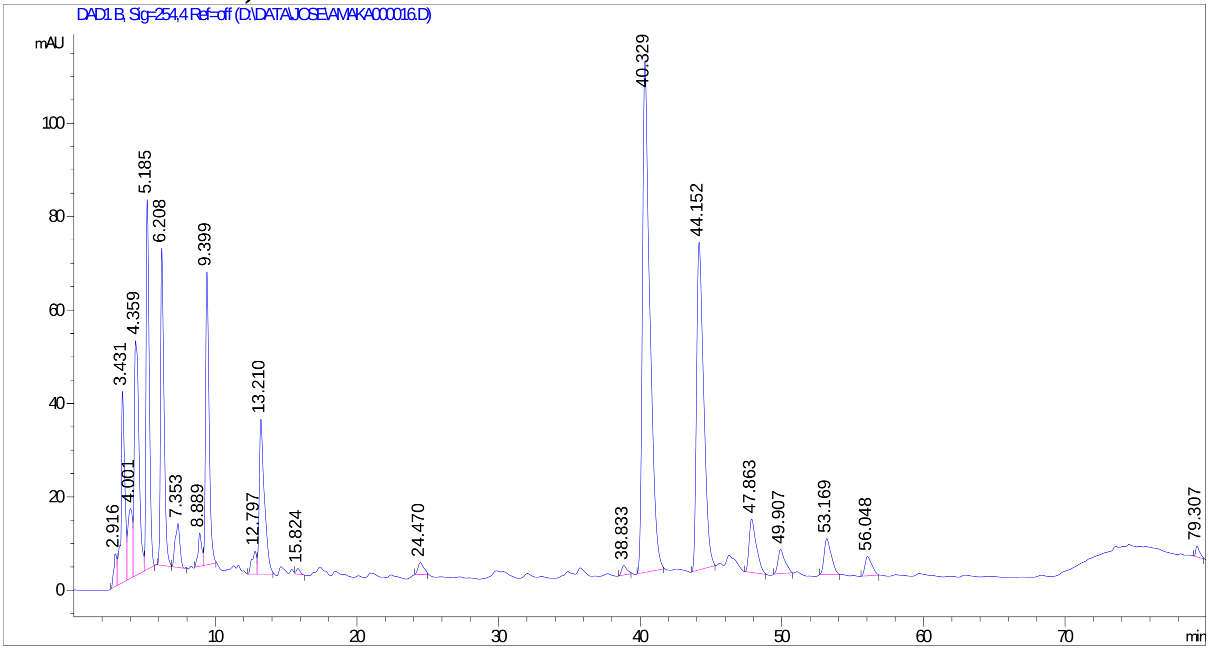

Supplementary Figure 17S. HPLC chromatogram (254 nm) of *O. gratissimum* (voucher no. SOP052011OG01)

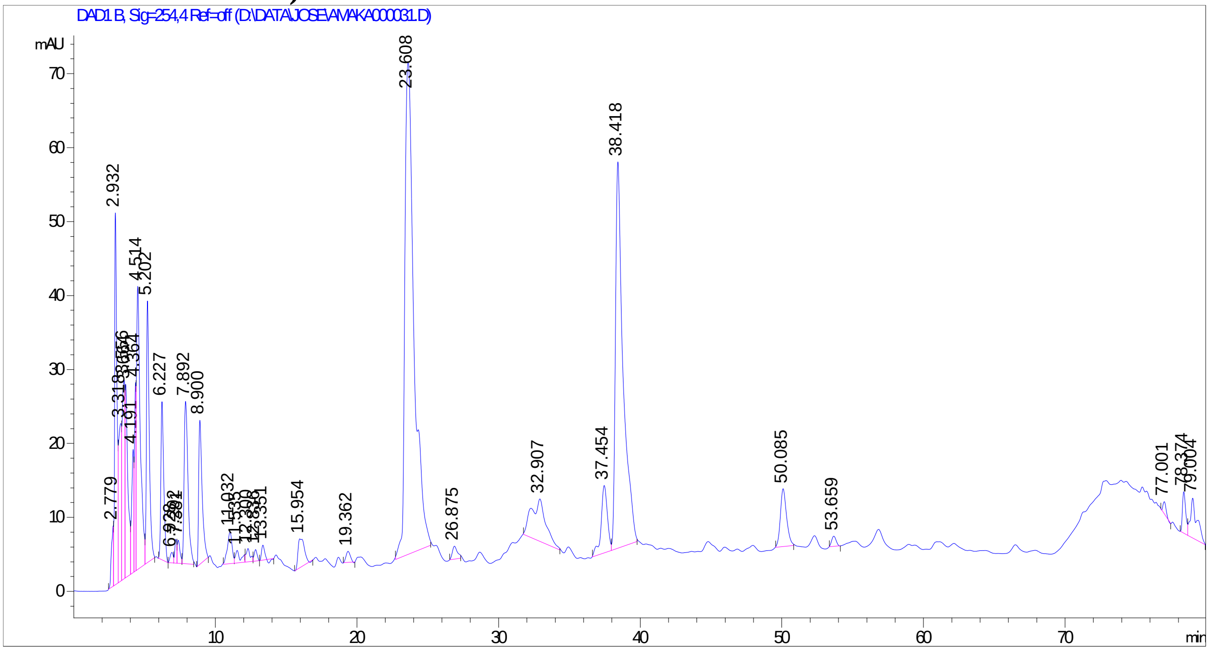

Supplementary Figure 18S. HPLC chromatogram (254 nm) of *P nitida* (voucher no. SOP052011PN01)

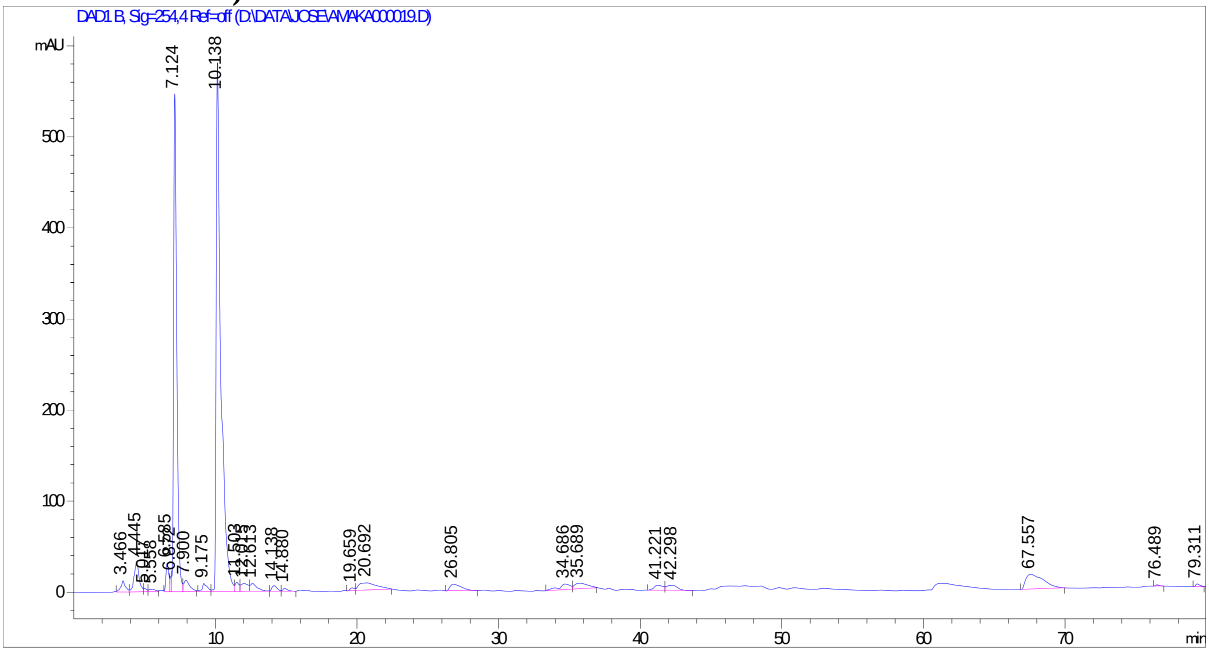

Supplementary Figure 19S. HPLC chromatogram (254 nm) of *R. vomitoria* (voucher no. SOP052011RV01)

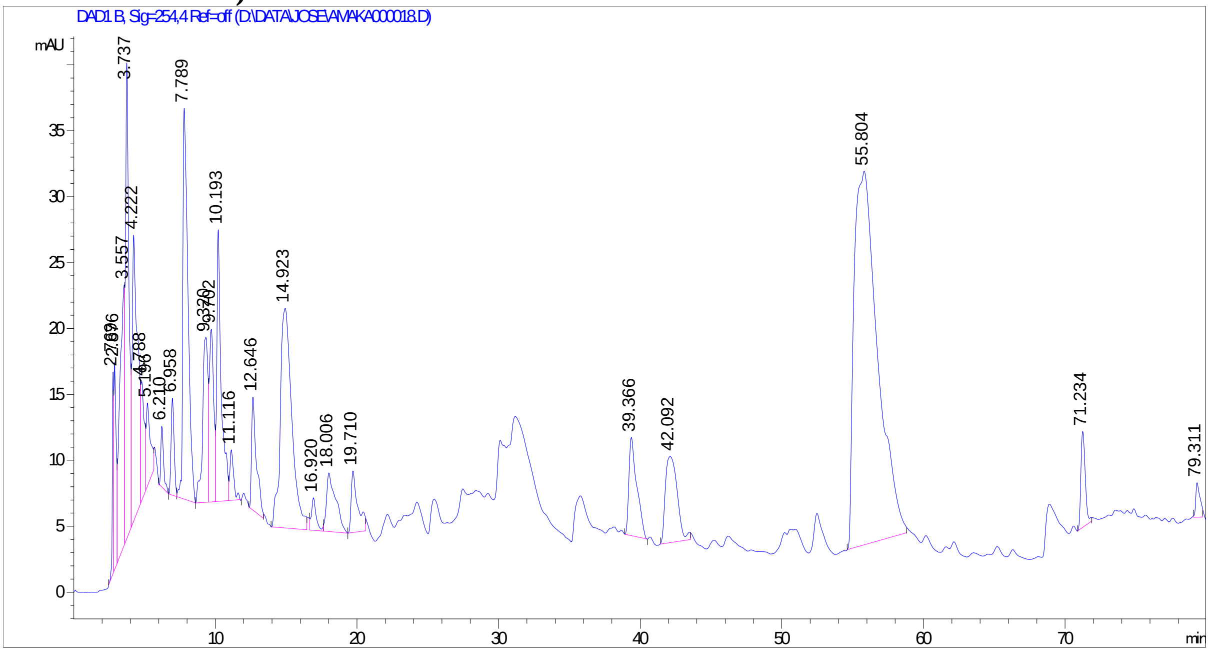

Supplementary Figure 20S. HPLC chromatogram (254 nm) of *S. dulcis* (voucher no. SOP052011SD01)

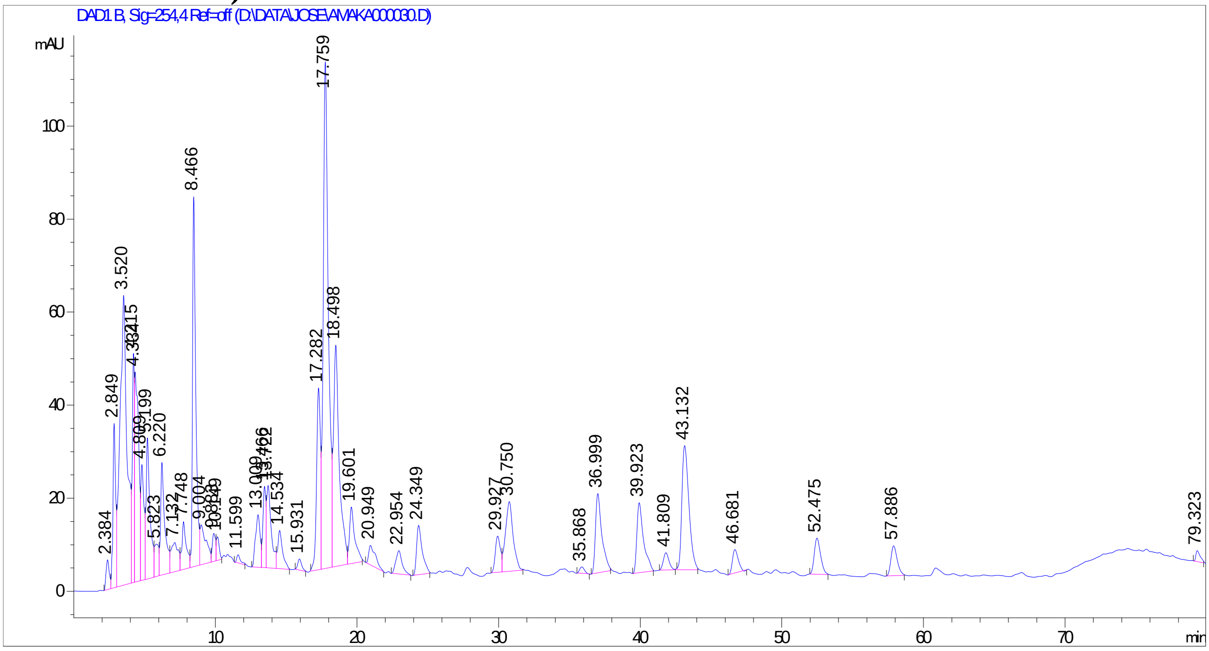

Supplementary Figure 21S. HPLC chromatogram (254 nm) of *S. longependulata* (voucher no. SOP052011SL01)

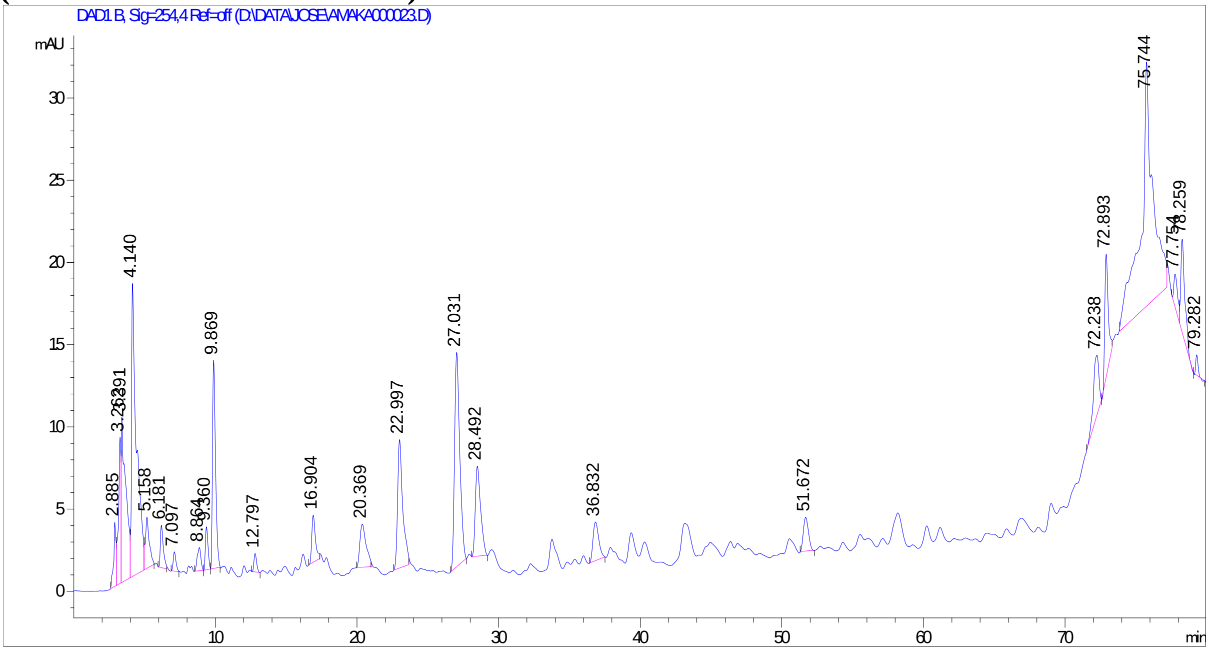

Supplementary Figure 22S. HPLC chromatogram (254 nm) of *S hispidus* (voucher no. SOP052011SH01)

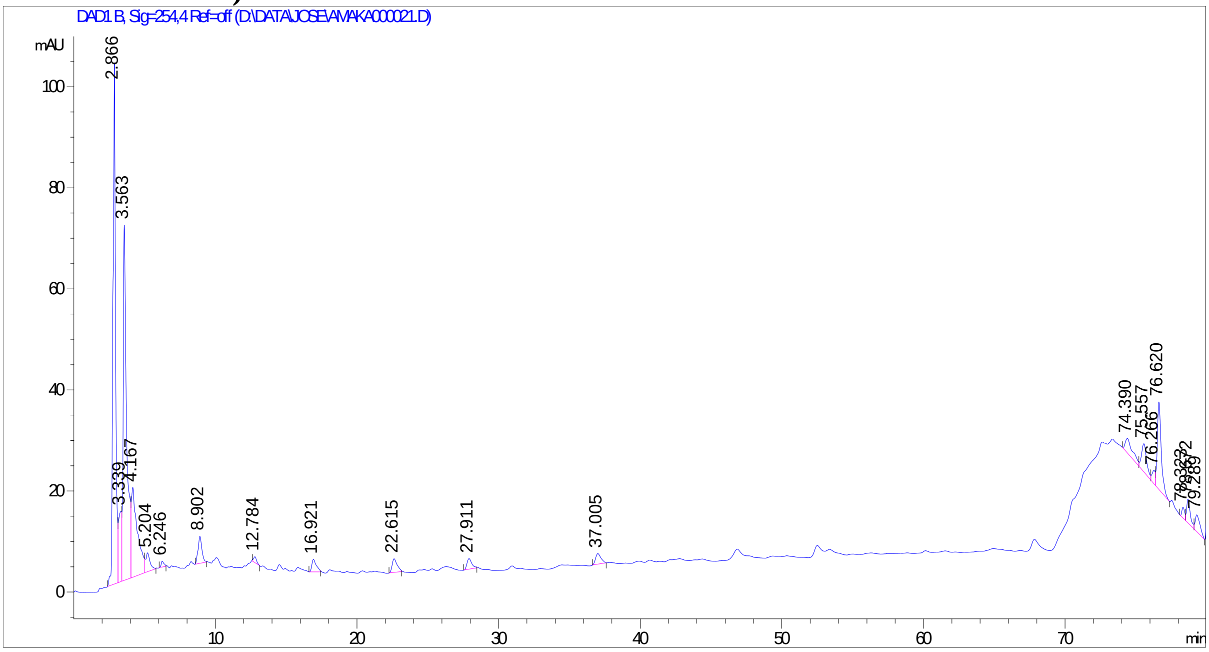

Supplementary Figure 23S. HPLC chromatogram (254 nm) of *S. guineense* (voucher no. SOP052011SG01)

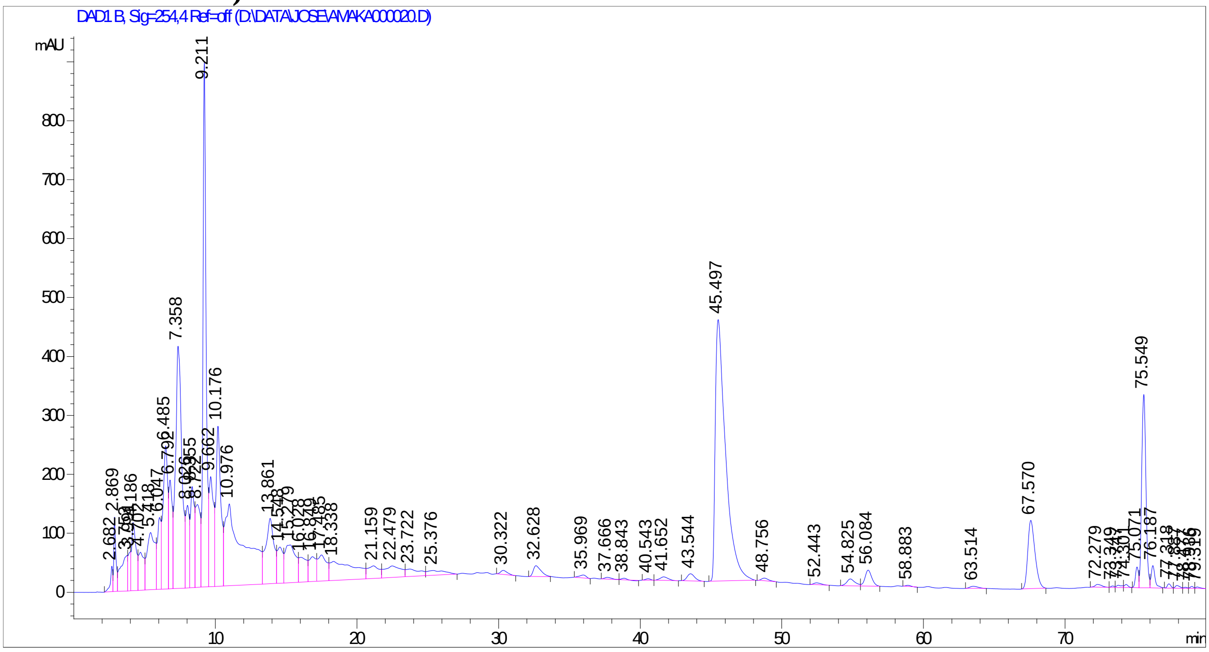

Supplementary Figure 24S. HPLC chromatogram (254 nm) of *Tamarindus indica* (voucher no. SOP052011TI01)

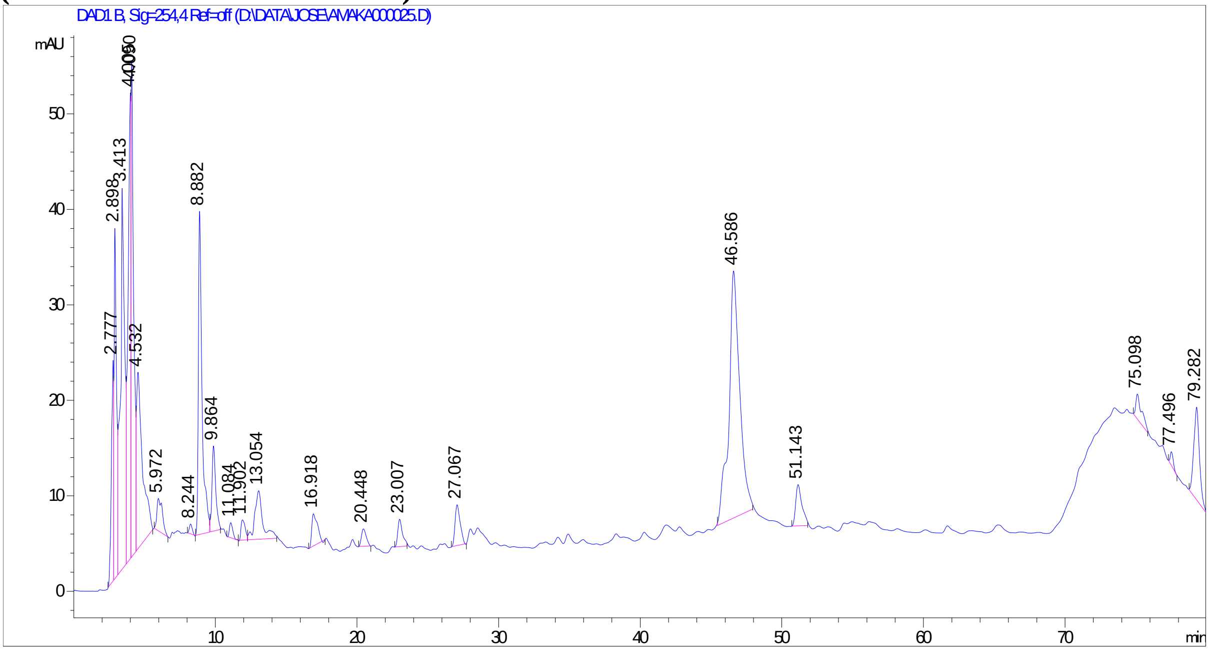

Supplementary Figure 25S. HPLC chromatogram (254 nm) of *T. avicennoides* (voucher no. SOP052011TA01)

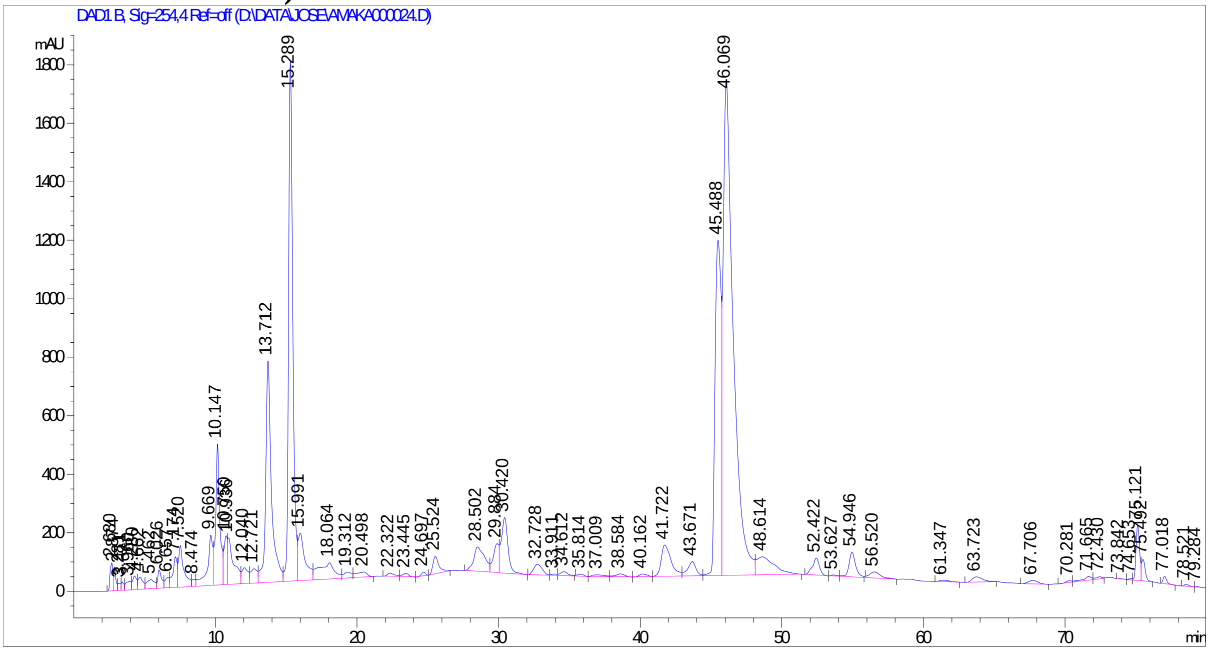

Supplementary Figure 26S. HPLC chromatogram (254 nm) of *T. bangwensis* (voucher no. SOP052011TB01)

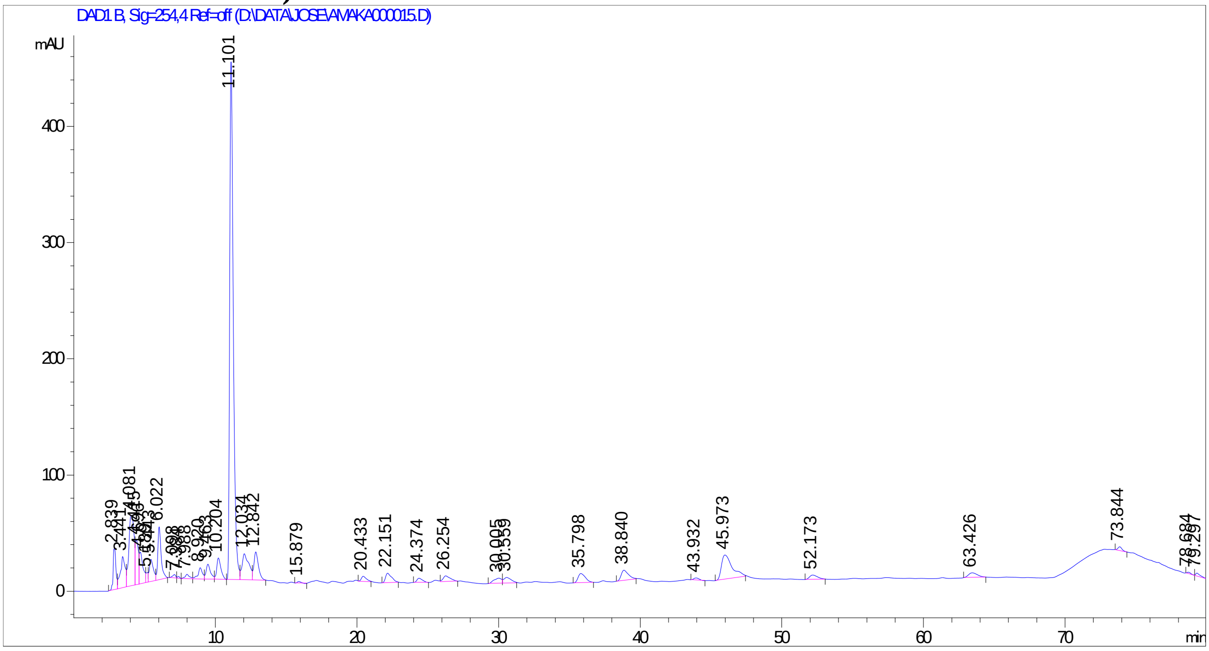

Supplementary Figure 27S. HPLC chromatogram (254 nm) of *V. amigdalina* (voucher no. SOP052011VA01)

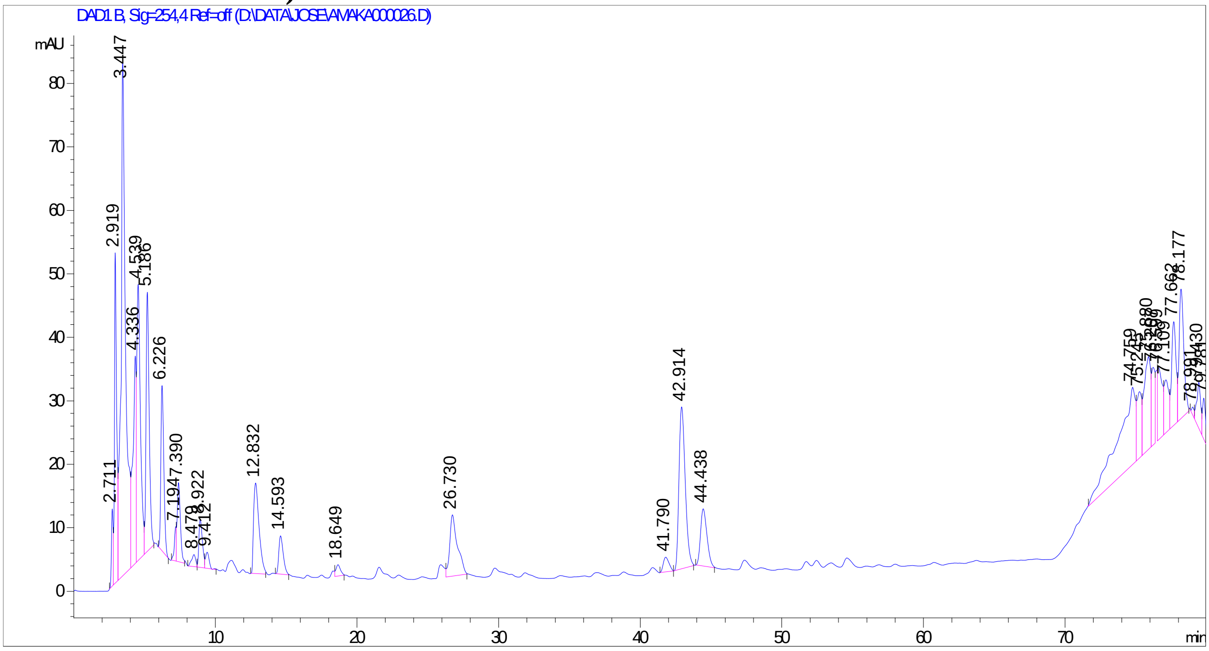

Supplementary Figure 28S. HPLC chromatogram (254 nm) of *X. americana* (voucher no. SOP052011XA01)

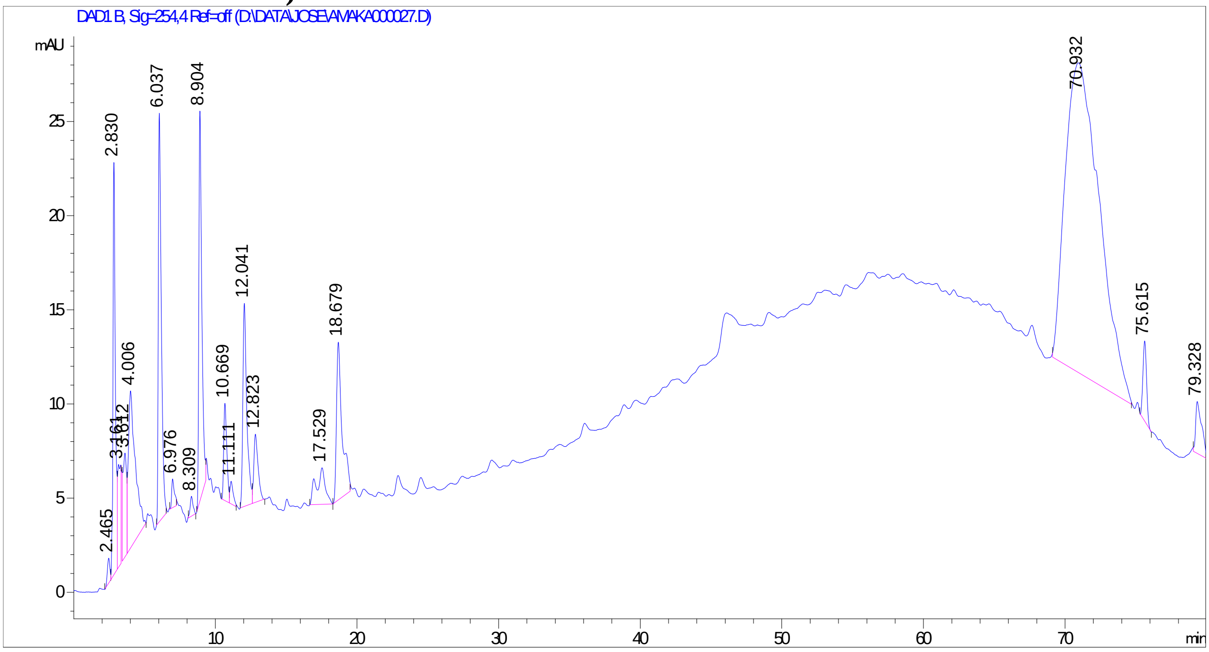

Supplementary Table 1S: Tabular summary of phenolic compounds present in plant samples

**Plant name (SampleID)**	**Phenolic standards potentially present in sample based on Rt and UV-spectra.**	**Phenolic standards undetected in the sample but known to be present in specie (Ref: Ezuruike and Prieto, 2014)**	**Other phenolic compounds known to be present in specie (Ref: Ezuruike and Prieto, 2014)**
A. boonei (AB1)	Quercitrin	None	None
A. senegalensis (AS1)	Epigallocatechin, Gallic acid	Catechin, Epicatechin, Quercetin, Rutin	Quercitin-3-*O*-glucoside (Isoquercetin)
A. leiocarpus (AL1)	Epigallocatechin, Gallic acid, Ellagic acid	None	Castalagin, Chlorogenic acid, Flavogallonic acid, *p*-Coumaric acid, Protocatechuic acid (3,4-dihydroxybenzoic acid),
A. djalonensis (AD1/AD2)	None	None	None
A. repenns (AR1)	None	None	None
B. ferruginea (BS1)	Epicatechin, Gallicacid	Kaempferol, Quercitrin, Vitexin	Apigenin, Ferrugin (Tri-O-methylmyricetin), Gallocatechin-(4'-O-7)-epigallocatechin, Isoquercetin, Myricetin, Myricetin- 3-rhamnoside, Quercetin-3-neohesperidoside, Tetra-O- methylmyricetin, Rutisin (Tetra-O-methylquercetin), 3-O-methylquercetin
C. sieberiana (CS1/CS2)	None Epicatechin	Quercitrin (Quercetin-3-*O*-rhamnoside)	Epiafzelechin
C. filiformis (CF1)	Galli cacid	Rutin (Quercetin-3-*O*-rutinoside)	Isorhamnetin, Isorhamnetin-3-*O*-β-glucoside,Isorhamnetin-3-*O*-rutinoside, Isorhamnetin-3-*O*-robinobioside, Quercetin-3-*O*-robinobioside, Isovanillin, Kaempferol-3-*O*-robinobioside, Vanillin
D. ogea(DO1)	Epicatechin, Gallicacid	None	None

Plant name (SampleID)	Phenolic standards potentially present in sample based on Rt and UV-spectra.	Phenolic standards undetected in the sample but known to be present in specie (Ref: Ezuruike and Prieto, 2014)	Other phenolic compounds known to be present in specie (Ref: Ezuruike and Prieto, 2014)
G. latifolium(GL1)	Caffeic acid, Vitexin rhamnoside	None	None
I. doka (ID1)	None	None	None
K .ivorensis(KS1/KS3)	None	None	None
K. senegalensis(KS2)	None	Catechin, Quercitrin, Rutin	Procyanidin
M. whitei(MW1)	None	None	Isovanillin (2-hydroxy-4-methoxybenzaldehyde),Vanillin(3- methoxy-4-hydroxybenzaldehyde), 6-methoxy-7- hydroxy coumarin, 6-methoxy-7,8-dihydroxycoumarin
M.oleifera(MO1)	None	None	Chlorogenicacid, Isoquercetin, Kaempferol glycoside, Rhamnetin, Vanillin
O. gratissimum (OG1	Caffeic acid	Isovitexin, Rutin, Vitexin	Apigenin-7-*O*-glucosdie, Caffeoyl derivatives, Cichoric acid, Circiliol, Cirsimaritin, Isoquercetin, Isothymusin, Kaempferol-3-*O*-rutinoside, Luteolin-5-*O*-glucoside, Luteolin-7-*O*-glucoside, Nevadensin Rosmarinic acid Vicenin-2
P. nitida (PN1)	None	None	None
R. vomitoria (RV1)	Gallic acid	None	Apigenin rhamnoside,Naringenin
R. dulcis(SD1)	Vitexin	Luteolin, Quercetin	Apigenin, Hispidulin, *p*-Coumaricacid
S. longipedunculata(SL1)	None	Gallic acid, Caffeic acid, Epicatechin, Quercetin, Rutin	Apigenin, Cinnamic acid, Chlorogenic acid, p-Coumaric acid, Sinapic acid, 3,4,5-tri-O-caffeoylquinic acid, 4,5-di-O-caffeic acid
S.hispidus(SH1)	None	None	None

Supplementary Materials

Plant name (SampleID)	Phenolic standards potentially present in sample based on Rt and UV-spectra.	Phenolic standards undetected in the sample but known to be present in specie (Ref: Ezuruike and Prieto, 2014)	Other phenolic compounds known to be present in specie (Ref: Ezuruike and Prieto, 2014)
S. guineense(SG1)	Gallic acid, Rutin[1]	None	None
**T. indica (TI1)**	None	Luteolin,Vitex in	Apigenin, Naringenin, Procyanidin B2,Vanillin
**T. bangwensis (ML1)**	Gallicacid	Catechin, Epicatechin, Rutin	Catechin-3-O-rhamnoside,Catechin-7-O-rhamnoside, Epicatechin gallate, Peltatoside, Quercetin-3-O-β-D-glucopyranoside,4-methoxy-catechin-7-O-rhamnoside
**T. avicennioides (TA1)** §	None	Ellagic acid	Ellagic acid rhamnoside, Flavo-gallonic acid, Punicalagin (Shuaibu et al., 2008) gallic acid (Ekong and
**V. amygdalina (VA1)**	None	Luteolin, Rutin	Apigenin glucuronide, Caffeoylquinic acid, Chlorogenic acid, Luteolin-7-O-glucosdie, Luteolin-7-O-glucuronide, Luteolin- 7-O-rutinoside, Luteolin-4-O-rutinoside (Idemudia, 1967)
**X. americana (XA1)**	Gallic acid	Quercetin, Quercitrin	Kaempferol-3-O-(6”-gallyolyl)-β-glucopyranoside,Quercetin-3-O-(6”-gallyolyl)-β-glucopyranoside, Quercetin-3-O-β-xylopyranoside

The compounds highlighted in bold have not been yet reported in the plant species.
§ TA1 isn’t reviewed in {Ezuruike and Prieto, 2014) so the references for its identified phenolic compounds are included in the table

End of Supplementary Materials

[1] Tankeu, F.N., Pieme, C.A., Biapa Nya, C.P. et al. BMC Complement Altern Med (2016) 16: 315. https://doi.org/10.1186/s12906-016-1263-1
